# Tumor Exosomal ENPP1 Hydrolyzes cGAMP to Inhibit cGAS‐STING Signaling

**DOI:** 10.1002/advs.202308131

**Published:** 2024-03-18

**Authors:** Yu An, Jinchao Zhu, Qihui Xie, Jianzhou Feng, Yanli Gong, Qian Fan, Jiao Cao, Zhi Huang, Weixiong Shi, Qingyuan Lin, Lingling Wu, Chaoyong Yang, Tianhai Ji

**Affiliations:** ^1^ Department of Pathology Shanghai Ninth People's Hospital Shanghai Jiao Tong University School of Medicine Shanghai 200011 P. R. China; ^2^ State Key Laboratory of Oral & Maxillofacial Reconstruction and Regeneration Key Laboratory of Oral Biomedicine Ministry of Education Hubei Key Laboratory of Stomatology School & Hospital of Stomatology Wuhan University Wuhan 430070 P. R. China; ^3^ Institute of Molecular Medicine Renji Hospital Shanghai Jiao Tong University School of Medicine Shanghai 200127 P. R. China; ^4^ The MOE Key Laboratory of Spectrochemical Analysis and Instrumentation State Key Laboratory of Physical Chemistry of Solid Surfaces, Department of Chemical Biology, College of Chemistry and Chemical Engineering Xiamen University Xiamen 361005 P. R. China

**Keywords:** cGAS‐STING signaling, immune escape, tumor exosomal ENPP1

## Abstract

To evade immune surveillance, tumor cells express ectonucleotide pyrophosphatase phosphodiesterase 1 (ENPP1) on the surface of their membrane, which degrades extracellular cyclic GMP‐AMP (cGAMP), thereby inhibiting the cyclic GMP‐AMP synthase (cGAS) stimulator of interferon gene (STING) DNA‐sensing pathway. To fully understand this tumor stealth mechanism, it is essential to determine whether other forms of ENPP1 with hydrolytic cGAMP activity also are present in the tumor microenvironment to regulate this innate immune pathway. Herein, it is reported that various tumor‐derived exosomes carry ENPP1, and can hydrolyze synthetic 2′3′‐cGAMP and endogenous 2′3′‐cGAMP produced by cells to inhibit cGAS‐STING pathway in immune cells. Moreover, tumor exosomal ENPP1 also can hydrolyze 2′3′‐cGAMP bound to LL‐37 (an effective transporter of 2′3′‐cGAMP) to inhibit STING signaling. Furthermore, high expression of ENPP1 in exosomes is observed isolated from human breast and lung cancer tissue, and tumor exosomal ENPP1 inhibited the immune infiltration of CD8+ T cells and CD4+ T cells. The results elucidate the essential function of tumor exosomal ENPP1 in the cGAS‐STING pathway, furthering understanding of the crosstalk between the tumor cells and immune system.

## Graphical abstract

ENPP1 proteins are secreted by various tumor cells to the extracellular space via the exosomes and ENPP1 highly enriched in these exosomes. To evade immune surveillance, tumor exosomal ENPP1 can effectively hydrolyze extracellular 2′3′‐cGAMP or 2′3′‐cGAMP bound to LL‐37 (an effective transporter of 2′3′‐cGAMP) to inhibit cGAS‐STING pathway in immune cells.

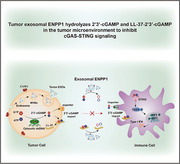

## 1 Introduction

The cGAS‐STING pathway is an essential, innate immune response pathway that plays a major role in autoimmune diseases and cancer.^[^ ^1^, ^2^, ^3^ ^]^ Mechanistically, cyclic GMP‐AMP synthase (cGAS), as a cytosolic DNA sensor, can first recognize double‐stranded DNA (dsDNA) from invading microbial pathogens or impaired cells and then synthesize 2′3′‐cyclic GMP‐AMP (2′3′‐cGAMP), using adenosine triphosphate (ATP) and guanosine triphosphate (GTP) as substrates.^[^ ^4^ ^]^ 2′3′‐cGAMP, as an endogenous second messenger, binds to the stimulator of interferon genes (STING) protein to recruit tank‐binding kinase 1 (TBK1) protein and activate interferon regulatory factor IRF3.^[^ ^4^ ^]^ Phosphorylated IRF3 then enters the nucleus and leads the production of type I interferons (IFNs) to initiate innate immune response for anticancer and antiviral.^[^ ^4^ ^]^ To enhance the host innate immune response, 2′3′‐cGAMP also can be exported extracellularly to activate cGAS‐STING signaling in bystander cells.^[^ ^5^ ^]^ Indeed, 2′3′‐cGAMP, as an anionic hydrophilic molecule, is transferred between cells dependent on virus particles, cell membrane channels, the SLC46A family of solute carriers, engineered transmembrane (TM)‐deficient STING protein, or antimicrobial peptides.^[^ ^6^, ^7^, ^8^, ^9^, ^10^, ^11^, ^12^, ^13^, ^14^, ^15^ ^]^ As key factors in the cGAS‐STING pathway, knowing the fate of extracellular cGAMP and transporter‐cGAMP complexes is necessary.

Chromatin instability (CIN) is prevalent in human tumors, and tumor cells sense cytosolic dsDNA from micronuclei to trigger the innate immune response.^[^ ^16^ ^]^ To survive, cancer cells have evolved to mitigate the effects of cGAS‐STING activation. Recent studies demonstrate that ectonucleotide pyrophosphatase/phosphodiesterase 1 (ENPP1), in cancer cell membrane‐bound form, can hydrolyze extracellular 2′3′‐cGAMP to block the attack of immune cells in the tumor microenvironment; ENPP1 inhibition and ionizing radiation (IR) synergistically promote extracellular cGAMP to delay tumor growth.^[^ ^5^, ^17^, ^18^ ^]^ In addition, ENPP1 can hydrolyze extracellular tumor‐derived 2′3′‐cGAMP into adenosine monophosphate (AMP) and guanosine monophosphate (GMP) to inhibit the cGAS‐STING signal pathway, and this ENPP1 inhibition induces an immune response and reduces tumor cell migration.^[^ ^16^ ^]^ These studies suggest that ENPP1 drives the immune evasion of tumors. At present, several pharmaceutical companies have developed inhibitors that target ENPP1. Among them, the ENPP1 inhibitor RBS2418, developed by Riboscience, has entered Phase I clinical trials.^[^ ^19^ ^]^ The clinical results in 11 different cancer types show that oral RBS2418 treatment has significant clinical benefits, which supports further clinical development of this novel immunotherapy agent.^[^ ^20^ ^]^ So far, ENPP1 is the only detectable protease that can hydrolyze immune transmitter 2′3′‐cGAMP. However, besides the ENPP1 protein distributed on the tumor cell membrane, it remains unknown whether other forms of ENPP1 protein that can hydrolyze 2′3′‐cGAMP are present in the tumor microenvironment.

Exosomes, which are lipid bilayer membrane vesicles (30–150 nm in diameter) secreted by numerous cell types, carry a variety of bioactive molecules (e.g., proteins and nucleic acids) of maternal cells to facilitate cell–cell communication.^[^ ^21^, ^22^ ^]^ Indeed, studies show that tumor‐derived exosomes are important communication mediators between tumor cells and not only immune cells, but also other nontumor bystander cells in the tumor microenvironment, and can regulate the immune response of the host.^[^ ^23^, ^24^, ^25^ ^]^ In particular, the role of tumor‐derived exosomes in regulating innate immune cGAS‐STING signaling gradually has been illucidated. For example, tumor cells treated with radiation have been shown to secrete exosomes carrying dsDNA to activate STING in immune cells.^[^ ^26^ ^]^ More recently, signal transducing adapter molecule 1 (STAM) has been shown to transport activated STING oligomers into exosomes and degrade STING oligomers to negatively regulate STING signaling.^[^ ^27^ ^]^ Although these studies suggest that exosomes play an important role in the regulation of the cGAS‐STING pathway, the molecular mechanism of cGAMP regulation by tumor‐derived exosomes remains unknown, which limits our full understanding of the regulatory mechanism of tumor‐derived exosomes in cGAS‐STING signaling pathway and tumor immune escape.

Against this backdrop, we report herein that various tumor cell‐derived exosomes carry ENPP1 proteins; indeed, ENPP1 is highly enriched in these exosomes. We found that tumor‐derived exosomes can assist free 2′3′‐cGAMP to active STING signaling. To weaken exosomes‐mediated immune enhancement, tumor exosomal ENPP1 further hydrolyze extracellular 2′3′‐cGAMP to inhibit cGAS‐STING pathway in immune cells. LL‐37, as an antimicrobial peptide expressed by multiple cell types, has been shown to be an effective transporter of 2′3′‐cGAMP and can enhance the immune response of cGAS‐STING signaling.^[^ ^15^ ^]^ Our study demonstrates also that tumor exosomal ENPP1 can effectively hydrolyze 2′3′‐cGAMP bound to LL‐37 to inhibit STING signaling in immune cells. Indeed, tumor exosomal ENPP1 may have a strong degradation activity for any form of 2′3′‐cGAMP, thus inhibiting cGAS‐STING signaling. Moreover, our study also demonstrates that tumor exosomal ENPP1 can hydrolyze endogenous 2′3′‐cGAMP produced by cells to inhibit cGAS‐STING pathway in bystander cells. Indeed, we observed that tumor exosomes exhibit high ENPP1 expression in tissue samples from lung cancer and breast cancer patients. Importantly, the expression level of tumor exosomal ENPP1 shown an inverse correlation with CD8+ T cells and CD4+ T cells infiltration, respectively. Overall, our study reveals a mechanism by which tumor exosomal ENPP1 inhibits cGAS‐STING signaling through the hydrolysis of 2′3′‐cGAMP. This highlights the important role of tumor‐derived exosomes in the innate immune response and deepens our understanding of the ENPP1‐mediated cGAS‐STING pathway.

## 2 Results

### 2.1 Tumor‐Derived Exosomes Express ENPP1 and Hydrolyze 2′3′‐cGAMP/LL‐37‐2′3′‐cGAMP

**Figure 1:**
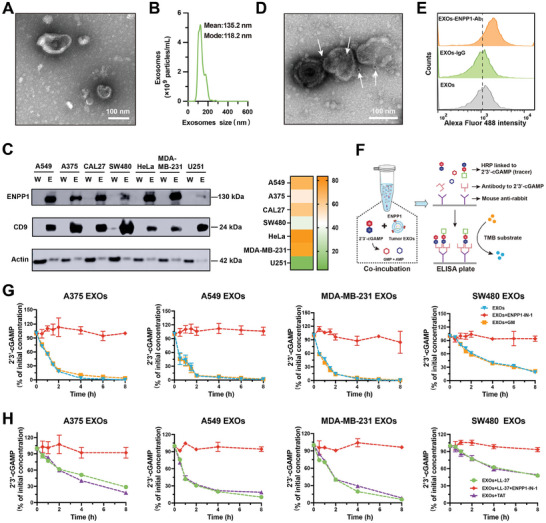
Tumor‐derived exosomes express ENPP1 and hydrolyze 2′3′‐cGAMP/LL‐37‐2′3′‐cGAMP. A) TEM image of A375 EXOs. B) Size distribution characterization of A375 EXOs by NTA. C) Western blot analysis of ENPP1 in whole cell lysate (W) and exosomes (E) from different tumor cell lines with the same amount of total protein (left). The relative intensity of ENPP1 in different tumor‐derived exosomes was analyzed via gray value of Western blot using Image J software (right). D) A representative TEM image of A375 EXOs immunogold‐labelled with anti‐ENPP1 antibodies. The arrows represent 5 nm gold particles. E) Flow cytometric analysis of ENPP1 expression in exosomes. F) Schematic of residual 2′3′‐cGAMP concentration determined by ELISA after hydrolysis of 2′3′‐cGAMP by tumor exosomal ENPP1. TMB, 3, 3′, 5, 5′‐tetramethylbenzidine; HRP, horseradish peroxidase. G) The percentage of residual 2′3′‐cGAMP to initial 2′3′‐cGAMP at different times of exosomes (0.5 mg mL^−1^) and 2′3′‐cGAMP (200 nM) co‐incubation. Exosomes from A375, A549, MDA‐MB‐231, and SW480 cells, with or without ENPP1‐IN‐1/GM treatment. H) The percentage of residual 2′3′‐cGAMP to initial 2′3′‐cGAMP at different times of exosomes (0.5 mg mL^−1^), LL‐37 (40 µg mL^−1^) and 2′3′‐cGAMP (200 nM) co‐incubation. TAT (40 µg mL^−1^) was selected as the negative control of LL‐37. Data are presented as mean ± SD (*n* = 2 independent experiments).

Exosomes were purified from various tumor cell lines of various tissue origin by differential centrifugation, and characterized using transmission electron microscopy (TEM) and nanoparticle tracking analysis (NTA) (**Figure** **1**A,B). Exosomes exhibited a typical cup shape morphology with a mean diameter of 135.2 nm. Western blot analysis revealed the presence of ENPP1 in exosomes derived from various human tumor cells lines, which included lung cancer A549, melanoma A375, oral cancer CAL27, colorectal cancer SW480, cervical cancer HeLa, breast cancer MDA‐MB‐231, and glioma U251 (Figure 1C). Moreover, the ENPP1 abundance in these exosomes was much higher than those in corresponding maternal cells (Figure 1C; Figure S1A, Supporting Information). Furthermore, ENPP1 was detected in exosomes derived from various mouse tumor cells (Figure S1B, Supporting Information), which indicates that ENPP1 may be expressed widely in tumor‐derived exosomes.

Tumor cell surface ENPP1 hydrolyzes extracellular 2′3′‐cGAMP to regulate innate immune response.^[^ ^5^ ^]^ Using immuno‐electron microscopy and flow cytometric analysis, we verified that ENPP1 was expressed on the surface of tumor‐derived exosomes (Figure 1D,E). To explore the hydrolytic capability of tumor exosomal ENPP1 to 2′3′‐cGAMP, a competition ELISA of 2′3′‐cGAMP assay was adopted (Figure 1F).^[^ ^28^ ^]^ We used (1) A375‐, A549‐, and MDA‐MB‐231 cell‐derived exosomes (A375 EXOs, A549 EXOs, and MDA‐MB‐231 EXOs) with high expression of ENPP1 and (2) SW480 cell‐derived exosomes (SW480 EXOs) with relatively low expression of ENPP1 as models. When incubating with tumor‐derived exosomes with high expression of ENPP1, 2′3′‐cGAMP was degraded rapidly and completely degraded at 4 h (Figure 1G). The SW480 EXOs with relatively low expression of ENPP1 also exhibited visible hydrolysis of 2′3′‐cGAMP after 0.5 h. When ENPP1‐IN‐1, a ENPP1 inhibitor,^[^ ^29^ ^]^ was introduced, 2′3′‐cGAMP hydrolysis was almost completely prevented. In contrast, control protease inhibitor gabexate mesylate (GM) had no influence on 2′3′‐cGAMP hydrolysis. These results verified effective hydrolytic capability of tumor exosomal ENPP1 to 2′3′‐cGAMP. Meanwhile, the human host defense peptide LL‐37 has been reported to be an effective transporter of 2′3′‐cGAMP into target cells to activate STING signaling by binding with 2′3′‐cGAMP to form LL‐37‐2′3′‐cGAMP.^[^ ^15^ ^]^ We further confirmed tumor exosomal ENPP1 also could hydrolyze 2′3′‐cGAMP in the form of LL‐37‐2′3′‐cGAMP (Figure 1H). In addition, we observed the hydrolytic capability of exosomal ENPP1 from mouse tumor cell lines to 2′3′‐cGAMP and LL‐37‐2′3′‐cGAMP (Figure S1C,D, Supporting Information). Collectively, these results verify that various tumor‐derived exosomes carried ENPP1 proteins, and tumor exsomal ENPP1 can hydrolyze 2′3′‐cGAMP and LL‐37‐2′3′‐cGAMP.

### 2.2 Tumor Exosomal ENPP1 Inhibits cGAS‐STING Signaling by Hydrolyzing 2′3′‐cGAMP

**Figure 2:**
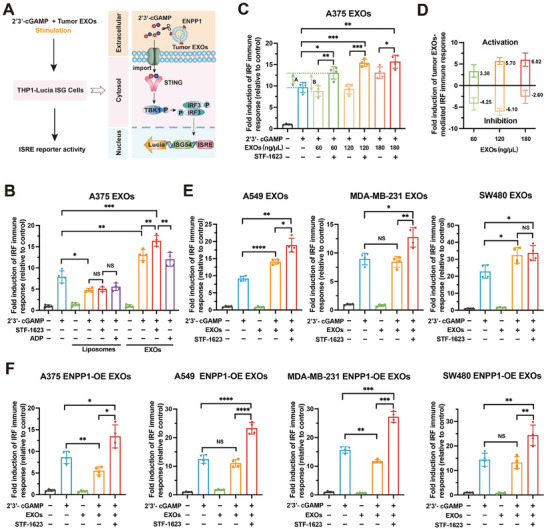
Tumor exosomal ENPP1 inhibits cGAS‐STING signaling by hydrolyzing 2′3′‐cGAMP. A) Schematic of tumor‐derived exosomes‐mediated immune response assay of THP1‐Lucia ISG cells. THP1‐Lucia ISG cells were treated with tumor‐derived exosomes and 2′3′‐cGAMP in the absence or presence of STF‐1623 for 24 h. Culture medium was collected for ISRE reporter activity analysis. B) ISRE reporter activity analysis of THP1‐Lucia ISG cells treated with 2′3′‐cGAMP (200 nM), A375 EXOs (150 ng µL^−1^), a combination of 2′3′‐cGAMP and A375 EXOs, a combination of 2′3′‐cGAMP, A375 EXOs and STF‐1623 (300 nM), or a combination of 2′3′‐cGAMP, A375 EXOs and ADP (300 nM) for 24 h. Meanwhile, liposomes (150 ng µL^−1^) were used as a control test. C) ISRE reporter activity analysis of THP1‐Lucia ISG cells treated with 2′3′‐cGAMP (200 nM) and different concentrations of A375 EXOs in the absence or presence of STF‐1623 (300 nM) for 24 h. D) Analysis of activation and inhibition of STING signaling mediated by different concentrations of exosomes. E) ISRE reporter activity analysis of THP1‐Lucia ISG cells treated with 2′3′‐cGAMP (200 nM), A549 EXOs (150 ng µL^−1^), a combination of 2′3′‐cGAMP and A549 EXOs, or a combination of 2′3′‐cGAMP, A549 EXOs and STF‐1623 (300 nM) (left) for 24 h. Similar experiments were performed using MDA‐MB‐231 EXOs (middle), or SW480 EXOs (right). F) ISRE reporter activity analysis of THP1‐Lucia ISG cells treated with 2′3′‐cGAMP (200 nM), A375 ENPP1‐OE EXOs (150 ng µL^−1^), a combination of 2′3′‐cGAMP and A375 ENPP1‐OE EXOs, or a combination of 2′3′‐cGAMP, A375 ENPP1‐OE EXOs and STF‐1623 (300 nM) for 24 h. Similar experiments were performed using A549 ENPP1‐OE EXOs, MDA‐MB‐231 ENPP1‐OE EXOs, SW480 ENPP1‐OE EXOs. Data are presented as mean ± SD (*n* = 4 independent experiments). All *p* values were determined by ANOVA. NS, *p* > 0.05, *p < 0.05, **p < 0.01, ***p < 0.001, ****p < 0.0001.

To facilitate detection of STING activation, human THP1‐Lucia ISG cells were stably integrated with luciferase reporter gene, under the control of five Interferon (IFN)‐Stimulated Response Elements (ISRE) fused to an ISG54 minimal promoter, whose ISRE reporter activity represents the activation of cGAS‐STING signaling.^[^ ^15^, ^30^ ^]^ As expected, either synthenic 2′3′‐cGAMP or LL‐37‐2′3′‐cGAMP increased ISRE reporter activity in a concentration‐dependent manner, and 200 nM was selected for the subsequent test in consideration of its physiological concentration (Figure S2A, Supporting Information).^[^ ^28^ ^]^ To reveal the regulation mechanism of tumor exosomal ENPP1 on cGAS‐STING signaling in immune cells, THP1‐Lucia ISG cells were stimulated with 2′3′‐cGAMP in the presence of tumor‐derived exosomes, in a serum‐free medium, to eliminate the interference of serum ENPP1 protein (**Figure** **2**A),^[^ ^5^ ^]^ and a series of controls were set. Compared with those treated with 2′3′‐cGAMP alone, THP1‐Lucia ISG cells, which were treated with both A375 EXOs and 2′3′‐cGAMP, exhibited higher levels of ISRE reporter activity (Figure 2B). In contrast, sole A375 EXOs did not affect the cGAS‐STING pathway of THP1‐Lucia ISG cells (Figure 2B; Figure S2B, Supporting Information). These results suggest that A375 EXOs could assist 2′3′‐cGAMP to activate cGAS‐STING signaling. As such, we adopted STF‐1623,^[^ ^5^, ^18^ ^]^ a phosphonate analog, instead of ENPP1‐IN‐1 as ENPP1 inhibitor, because the latter could affect the STING signaling in the presence of 2′3′‐cGAMP (Figure S2C, Supporting Information). Our synthesized STF‐1623 (Figure S2D, Supporting Information) and adenosine diphosphate (ADP, a negative control for STF‐1623) did not affect cGAS‐STING signaling with or without 2′3′‐cGAMP (Figure S2E,F, Supporting Information). When introducing STF‐1623 to the incubation process of 2′3′‐cGAMP and exosomes, although the ISRE reporter activity was enhanced (Figure 2B), no activity change for control ADP was observed (Figure 2B), which indicated that tumor exosomal ENPP1 inhibited cGAS‐STING pathway by hydrolyzing 2′3′‐cGAMP. We took liposomes as the control exosomes.^[^ ^31^ ^]^ The cGAMP‐induced ISRE reporter activities in THP1‐Lucia ISG cells revealed no statistically significant difference among treatments of incubating with either liposomes alone or in presence of STF1623 or ADP (Figure 2B; Figure S2G, Supporting Information), which highlights the important roles of tumor‐derived exosomes in regulating the cGAS‐STING signaling pathway.

To further define the relevance of the tumor‐derived exosomes and cGAS‐STING pathway, we investigated the effect of different concentrations of exosomes on the ISRE reporter activities. Disparity A represents the difference in ISRE reporter activity between THP1‐Lucia ISG cells treated with 2′3′‐cGAMP and those treated with combined 2′3′‐cGAMP, exosomes, and STF‐1623, which indicates the enhancement effect of tumor‐derived exosome assisted 2′3′‐cGAMP on the cGAS‐STING pathway (Figure 2C). Disparity B reflects the difference in ISRE reporter activity of THP1‐Lucia ISG cells treated with a combination of exosomes and 2′3′‐cGAMP with or without STF‐1623, which indicates the inhibitory effect of tumor exosomal ENPP1 on the cGAS‐STING pathway through hydrolysis of 2′3′‐cGAMP (Figure 2C). We observed that tumor exosomal ENPP1 can hydrolyze 2′3′‐cGAMP to inhibit cGAS‐STING signaling, regardless of the concentration of exosomes (Figure 2D). However, by comparing disparities A and B (Figure 2D), we observed that lower concentrations of exosomes exhibited negative regulation of cGAS‐STING signaling due to tumor exosomal ENPP1‐dominated hydrolysis of 2′3′‐cGAMP (Figure 2C,D). With increased exosome concentration, tumor‐derived exosomes exhibited positive regulation of cGAS‐STING signaling due to enhanced assistance of exosomes to 2′3′‐cGAMP (Figure 2C,D). In addition, exosomes from mouse tumor LLC‐1 cells exhibited a similar dual positive and negative regulation in the STING signaling of RAW‐Lucia ISG cells (Figure S2H, Supporting Information).^[^ ^32^, ^33^ ^]^ Taken together, these data demonstrate the dual function of A375 EXOs in the positive and negative regulation of the cGAS‐STING pathway—they assist 2′3′‐cGAMP in enhancing cGAS‐STING signaling, while exosomal ENPP1 hydrolyzes 2′3′‐cGAMP to inhibit the cGAS‐STING pathway.

Similar results were obtained for A549 EXOs and MDA‐MB‐231 EXOs (Figure 2E). These tumor‐derived exosomes also exhibited dual regulation in the cGAS‐STING pathway by both assisting 2′3′‐cGAMP to enhance STING signaling and hydrolyzing 2′3′‐cGAMP through ENPP1 to inhibit STING signaling. The inhibition of the pathway by SW480 exosomal ENPP1 was not evident, and we hypothesize that this is probably due to less ENPP1 protein being present on the exosomes (Figure 2E). To further exucidate the function of tumor exosomal ENPP1, A375, MDA‐MB‐231, A549, and SW480 ENPP1‐overexpressed (ENPP1‐OE) cell lines were created by stably expressing human ENPP1 (Figure S2I, Supporting Information). The exosomes secreted by these cells all overexpressed ENPP1 proteins (A375, MDA‐MB‐231, A549, and SW480 ENPP1‐OE EXOs; Figure S2J, Supporting Information). As expected, compared with the effect of STF‐1623 on exosome‐mediated immune response in Figure 2E, the tumor ENPP1‐OE EXOs‐mediated ISRE reporter activity was more upregulated in the presence of STF‐1623 (Figure 2F). These data confirm the powerful function of tumor exosomal ENPP1 in inhibiting the cGAS‐STING pathway through hydrolysis of 2′3′‐cGAMP.

### 2.3 Tumor Exosomal ENPP1 Inhibits cGAS‐STING Signaling by Hydrolyzing LL‐37‐2′3′‐cGAMP

**Figure 3:**
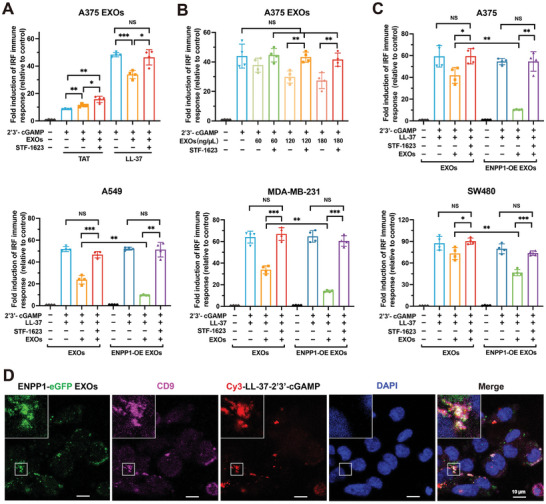
Tumor exosomal ENPP1 inhibits cGAS‐STING signaling by hydrolyzing LL‐37‐2′3′‐cGAMP. A) ISRE reporter activity analysis of THP1‐Lucia ISG cells treated with LL‐37 (40 µg mL^−1^)−2′3′‐cGAMP (200 nM), A375 EXOs (150 ng µL^−1^), a combination of LL‐37‐2′3′‐cGAMP and A375 EXOs, or a combination of LL‐37‐2′3′‐cGAMP complex, A375 EXOs and STF‐1623 (300 nM) for 24 h. Meanwhile, TAT (40 µg mL^−1^) was used as a control test. B) ISRE reporter activity analysis of THP1‐Lucia ISG cells treated with LL‐37 (40 µg mL^−1^)−2′3′‐cGAMP (200 nM) and different concentrations of A375 EXOs in the absence or presence of STF‐1623 (300 nM) for 24 h. C) ISRE reporter activity analysis of THP1‐Lucia ISG cells treated with LL‐37 (40 µg mL^−1^)−2′3′‐cGAMP (200 nM), a combination of LL‐37‐2′3′‐cGAMP complex and A375 EXOs (150 ng µL^−1^) in the absence or presence of STF‐1623 (300 nM), or a combination of LL‐37‐2′3′‐cGAMP and A375 ENPP1‐OE EXOs (150 ng µL^−1^) in the absence or presence of STF‐1623 (300 nM) for 24 h. Similar experiments were performed using A549 ENPP1‐OE EXOs, MDA‐MB‐231 ENPP1‐OE EXOs, SW480 ENPP1‐OE EXOs. Data are presented as mean ± SD (*n* = 4 independent experiments). All p values were determined by ANOVA. NS, *p* > 0.05, *p < 0.05, **p < 0.01, ***p < 0.001, ****p < 0.0001. D) The colocalization analysis of LL‐37‐2′3′‐cGAMP labeled Cy3, A375 EXOs labeled ENPP1‐eGFP and CD9 labeled Alexa Flour 647, an exosome marker, in THP‐1 cells. The fluorescence imaging was performed by the Leica TCS SP8 CARS using a 63X objective.

To investigate the role of tumor exosomal ENPP1 in the LL‐37‐2′3′‐cGAMP‐mediated cGAS‐STING signaling pathway, THP1‐Lucia ISG cells were treated with LL‐37‐2′3′‐cGAMP in presence of tumor‐derived exosomes, and corresponding controls were set. In constrast to tumor‐derived exosomes (150 ng µL^−1^) enhancing STING signaling induced by 2′3′‐cGAMP (Figure 2B), the same concentration of tumor‐derived exosomes induced a decrease in LL‐37‐mediated 2′3′‐cGAMP response (**Figure** **3**A). Moreover, when introducing STF‐1623 to the incubation process of LL37‐2′3′‐cGAMP and exosomes, the ISRE reporter activity was restored to the same level as when treated with only LL‐37‐2′3′‐cGAMP (Figure 3A). These results suggest that tumor‐derived exosomes did not assist LL‐37‐2′3′‐cGAMP to activate cGAS‐STING pathway, possibly due to the efficient transfer of LL‐37‐2′3′‐cGAMP toward bystander cells without any need of other transporter. Therefore, in the case of LL‐37‐2′3′‐cGAMP, tumor‐derived exosomes only inhibit the cGAS‐STING pathway by hydrolyzing LL‐37‐2′3′‐cGAMP through ENPP1. TAT, a cell‐penetrating peptide, unable to bind to 2′3′‐cGAMP, was employed as the control LL‐37 (Figure 3A).^[^ ^15^ ^]^ As expected, consistent with the results shown in Figure 2B, A375 EXOs exhibited the dual function of assisting 2′3′‐cGAMP to enhance the cGAS‐STING signaling pathway and hydrolyzing 2′3'‐cGAMP by surface ENPP1 to inhibit the pathway. This certainly suggests that tumor‐derived exosomes play different roles in 2′3′‐cGAMP or LL‐37‐2′3′‐cGAMP induced cGAS‐STING signaling. Indeed, unlike free 2′3′‐cGAMP (Figure 2C), LL‐37‐2′3′‐cGAMP‐induced ISRE reporter activity was not affected by tumor‐derived exosomes concentration in presence STF‐1623 (Figure 3B), which suggests further that tumor‐derived exosomes did not assist LL‐37‐2′3′‐cGAMP to activate cGAS‐STING pathway. Tumor‐derived exosomes with different concentrations only could downregulate STING signaling. These results reveal that tumor‐derived exosomes only can negatively regulate the cGAS‐STING signaling pathway induced by LL‐37‐2′3′‐cGAMP through hydrolyzing cGAMP by ENPP1. Therefore, we conclude that tumor‐derived exosomes play different roles in different modes of 2′3′‐cGAMP: both assist and degrade free 2′3′‐cGAMP to active or inhibit cGAS‐STING signaling pathways in a concentration‐dependance manner, yet only degrade LL‐37‐2′3′‐cGAMP to inhibit cGAS‐STING signaling pathways.

In addition, similar observations were made for A549 EXOs, MDA‐MB‐231 EXOs, and SW480 EXOs (Figure 3C), which suggests that these exosomal ENPP1 also hydrolyzed LL‐37‐2′3′‐cGAMP to inhibit cGAS‐STING signaling. ENPP1‐OE exosomes downgraded LL‐37‐2′3′‐cGAMP‐induced ISRE reporter activity, compared to exosomes derived from wild‐type cells (Figure 3C). This demonstrates the important role of tumor exosomal ENPP1 in inhibiting the cGAS‐STING signaling pathway. Moreover, exosomal ENPP1 from mouse tumor cells revealed inhibition of STING signaling in RAW‐Lucia ISG cells by hydrolyzed LL‐37‐2′3′‐cGAMP (Figure S3A, Supporting Information). To examine the interaction of tumor exosomal ENPP1 and LL‐37‐2′3′‐cGAMP, we obtained exosomes from ENPP1‐eGFP‐overexpressed cells (A375 ENPP1‐eGFP EXOs, Figure S3B, Supporting Information). A375 ENPP1‐eGFP EXOs exhibit a strong fluorescence signal (Figure S3C,D, Supporting Information). As expected, confocal microscopic imaging exhibited a co‐localization of ENPP1‐eGFP EXOs, Cy3‐LL‐37‐2′3′‐cGAMP, and CD9, an exosome marker, in THP‐1 cells (Figure 3D). This result provides clear evidence for the physical interaction between tumor‐derived exosomes and LL‐37‐2′3′‐cGAMP. Collectively, all results verified that tumor exosomal ENPP1 plays an important role in the hydrolysis 2′3′‐cGAMP bound to LL‐37 to inhibit cGAS‐STING signaling.

### 2.4 Tumor Exosomal ENPP1 Hydrolyzes Endogenous 2′3′‐cGAMP Produced by Cells to Inhibit cGAS‐STING Signaling in Bystander Cells

**Figure 4:**
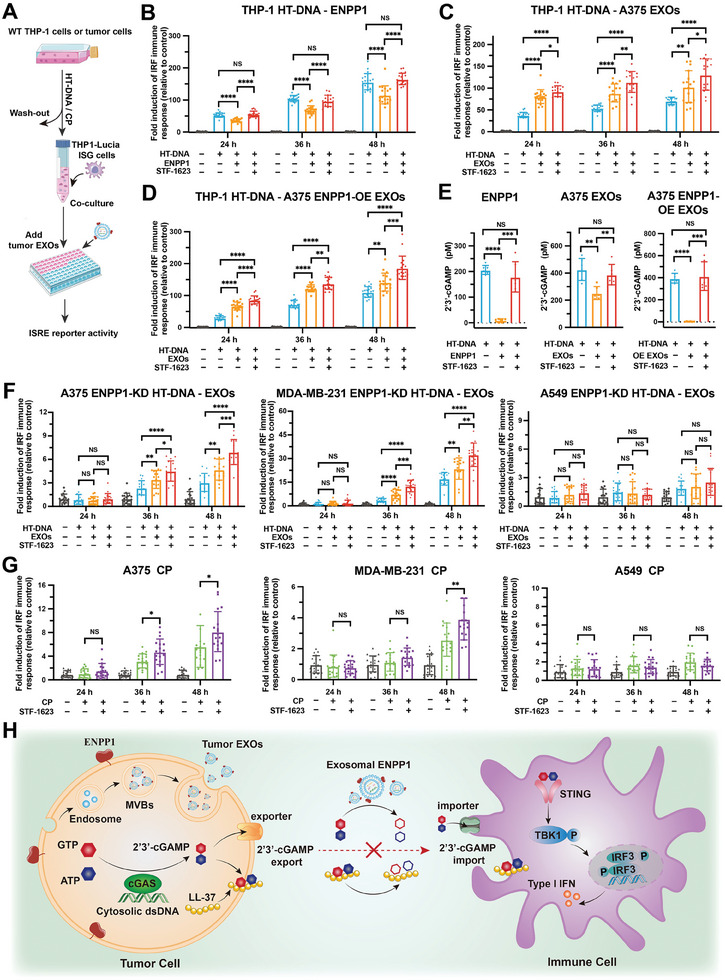
Tumor exosomal ENPP1 hydrolyzes endogenous 2′3′‐cGAMP produced by cells and inhibits cGAS‐STING signaling in bystander cells. A) Schematic of co‐culture system using WT THP‐1 cells for 2′3′‐cGAMP production and THP1‐Lucia ISG cells for immune response. WT THP‐1 cells were treated with HT‐DNA (50 ng mL^−1^) for 6 h, after which were removed and THP1‐Lucia ISG cells were co‐cultured for up to 48 h with or without STF‐1623 (300 nM) in presence of ENPP1 protein or tumor‐derived exosomes. Culture medium with different time was collected for ISRE reporter activity analysis. HT‐DNA or CP‐treated human tumor cells were co‐cultured with THP1‐Lucia ISG cells using a similar protocol. B–D) ISRE reporter activity analysis of THP1‐Lucia ISG cells co‐cultured with HT‐DNA‐treated WT THP‐1 cells for up to 48 h with or without STF‐1623 (300 nM) in presence of ENPP1 protein (0.4 ng µL^−1^) (B), A375 EXOs (40 ng µL^−1^) (C) or A375 ENPP1‐OE EXOs (40 ng µL^−1^) (D). E) Concentration measurement of extracellular 2′3′‐cGAMP in cells co‐cultured for 24 h by 2′3′‐cGAMP ELISA Kit (*n* = 6 independent experiments). F) ISRE reporter activity analysis of THP1‐Lucia ISG cells co‐cultured with HT‐DNA (50 ng mL^−1^)‐treated different ENPP1 knockdown (KD) tumor cells for up to 48 h with or without STF‐1623 (300 nM) in presence of A375 EXOs (40 ng/µL), MDA‐MB‐231 EXOs (40 ng µL^−1^), or A549 EXOs (40 ng µL^−1^). G) ISRE reporter activity analysis of THP1‐Lucia ISG cells co‐cultured with CP (5 µM)‐treated different human tumor cells for up to 48 h with or without STF‐1623 (300 nM). Data are presented as mean ± SD (*n* = 18 independent experiments). All p values were determined by ANOVA. NS, *p* > 0.05, *p < 0.05, **p < 0.01, ***p < 0.001, ****p < 0.0001. H) A mechanism model of tumor exosomal ENPP1‐mediated cGAS‐STING signaling pathway by hydrolyzing 2′3′‐cGAMP and LL‐37‐2′3′‐cGAMP in the tumor microenvironment.

To determine the effect of tumor exosomal ENPP1 on the cGAS‐STING pathway induced by endogenous 2′3′‐cGAMP, we performed a co‐culture experiment.^[^ ^34^ ^]^ THP‐1 cells, which barely express ENPP1 proteins, were used as models for endogenous 2′3′‐cGAMP production, which could avoid interference from endogenous ENPP1.^[^ ^17^ ^]^ Because dsDNA can stimulate cells to produce 2′3′‐cGAMP, which is actively exported to the extracellular space,^[^ ^5^ ^]^ we treated wild‐type (WT) THP‐1 cells with herring testicular DNA (HT‐DNA) to induce the production of endogenous 2′3′‐cGAMP.^[^ ^35^ ^]^ After changing the fresh serum‐free medium, the cells were co‐cultured with THP1‐Lucia ISG cells together with ENPP1 protein or exosomes treatment for cGAS‐STING signaling monitoring (**Figure** **4**A). As a positive control, we observed that ENPP1 protein could only hydrolyze 2′3′‐cGAMP produced by WT THP‐1 cells and downregulate ISRE reporter activity of the bystander THP1‐Lucia ISG cells (Figure 4B; Figure S4A, Supporting Information). Consistent with our previous results of tumor‐derived exosomes‐mediated synthetic 2′3′‐cGAMP response (Figure 2B), tumor‐derived exosomes enhanced endogenous 2′3′‐cGAMP‐induced STING signaling in THP1‐Lucia ISG cells (Figure 4C). Moreover, the exosomal ENPP1 blockade further increased the ISRE reporter activity. These results demonstrate the dual positive and negative regulation of tumor‐derived exosomes in the STING signaling of bystander cells. These exosomes can assist endogenous 2′3′‐cGAMP to enhance STING signaling and hydrolyze 2′3′‐cGAMP through surface ENPP1 to inhibit STING signaling. Indeed, ENPP1‐OE EXOs showed a stronger capability to inhibit cGAS‐STING signaling than that of exosomes from WT tumor cells (inhibition rate 22.0% versus 12.4% in 24 h) due to the high expression of ENPP1 (Figure 4D).

To determine whether tumor exosomal ENPP1 directly regulates the extracellular 2′3′‐cGAMP level, we measured the concentration of extracellular 2′3′‐cGAMP in cells co‐cultured for 24 h by 2′3′‐cGAMP ELISA Kit. The extracellular 2′3′‐cGAMP level was higher in the co‐culture system with STF‐1623 treatment than that without STF‐1623 treatment (Figure 4E), which indicates that the cGAMP hydrolase activity of tumor exosomal ENPP1 was blocked by STF‐1623. As expected, tumor exosomal ENPP1 overexpression reduced the extracellular 2′3′‐cGAMP level. These results provide additional evidence that tumor exosomal ENPP1 can efficiently hydrolyze extracellular 2′3′‐cGAMP produced by cells to negatively regulate STING signaling in bystander cells. To verify whether the regulation of tumor exosomal ENPP1 on immune response induced by 2′3′‐cGAMP is dependent on STING, STING inhibitor STING‐IN‐2^[^ ^36^ ^]^ was introduced into the co‐culture system to inhibit STING in THP1‐Lucia ISG cells. Indeed, ISRE reporter activity of THP1‐Lucia ISG cells did not differ in a statistically different way upon tumor‐derived exosomes and STF‐1623 treatment and remained consistent with the control (Figure S4B, Supporting Information). This result suggests that tumor exosomal ENPP1‐mediated immune response induced by 2′3′‐cGAMP is dependent on host STING. In summary, these results demonstrate that tumor exosomal ENPP1‐mediated extracellular 2′3′‐cGAMP hydrolysis inhibits cGAS‐STING signaling in bystander cells.

To test the role of tumor exosomal ENPP1 in the tumor immune microenvironment, we investigated the regulation of tumor exosomal ENPP1 on immune response induced by endogenous 2′3′‐cGAMP in a co‐culture system of tumor cells and immune cells. To eliminate the interference of ENPP1 protein on the surface of tumor cells, ENPP1 of tumor cells (A375, MDA‐MB‐231, and A549) was knocked down using shRNA against ENPP1 (Figure S4C, Supporting Information). Then, we treated ENPP1 knockdown tumor cells (A375, MDA‐MB‐231 and A549 ENPP1‐KD) with HT‐DNA (50 ng mL^−1^) and subsequently co‐cultured them with THP1‐Lucia ISG cells together with tumor‐derived exosomes treatment (Figure 4A). In A375 and MDA‐MB‐231 ENPP1‐KD cells, we found that tumor‐derived exosomes enhanced endogenous 2′3′‐cGAMP‐induced STING signaling in THP1‐Lucia ISG cells. Moreover, tumor exosomal ENPP1 disrupts 2′3′‐cGAMP transfer from paracrine tumor to bystander THP1‐Lucia ISG cells, which inhibited cGAS‐STING signaling in immune cells (Figure 4F). These results demonstrate that tumor‐derived exosomes play both positive and negative roles in regulating cGAS‐STING in the tumor immune microenvironment. However, in A549 ENPP1‐KD cells, we observed no change in ISRE reporter activity upon exosomes and STF‐1623 treatment, probably because the A549 cell type was insensitive to 50 ng mL^−1^ HT‐DNA and, therefore, did not produce enough 2′3′‐cGAMP to sufficiently activate the cGAS‐STING pathway in THP1‐Lucia ISG cells (Figure 4F). To further directly observe the role of endogenous exosomes produced by tumor cells in the tumor microenvironment, we did not add additional exosomes in the co‐culture system of wild tumor and immune cells. In A375 and MDA‐MB‐231 cell types, the ENPP1 blockade increased the ISRE reporter activity (Figure S4D, Supporting Information). Similar results were seen in the co‐culture system of ENPP1‐overexpressed A375 cells and THP1‐Lucia ISG cells (Figure S4D, Supporting Information). ENPP1 inhibitors (STF‐1623) should, in principle, inhibit both tumor cellular ENPP1 and tumor exosomal ENPP1. We next sought to directly link the tumor exosomal ENPP1 and cGAS‐STING signaling by introducing GW4869,^[^ ^37^ ^]^ an inhibitor of exosome synthesis and secretion, into a co‐culture system. Unfortunately, GW4869 had a serious effect on the cGAS‐STING pathway of THP1‐Lucia cells and could not be employed for the study of exosomes and immune response (Figure S4E, Supporting Information). Although it is still an open question whether tumor celluar ENPP1 or tumor exosomal ENPP1 has a bigger role, tumor cells secreting exosomes into the tumor microenvironment are well established. Collectively, based on the results of our extensive in vitro experiments, tumor exosomal ENPP1 definitely plays a crucial role in the regulation of cGAS‐STING signaling pathway.

To expand the role of ENPP1 in tumor immunotherapy, we characterized the modulation of immune cells by tumor cells that produced 2′3′‐cGAMP under stimulation of the chemotherapeutic drug cisplatin.^[^ ^38^ ^]^ The combination of cisplatin (CP, 5 µM)^[^ ^39^ ^]^ and STF‐1623 (300 nM) in A375, MDA‐MB‐231, and ENPP1‐overexpressed A375 cell types effectively enhanced the ISRE reporter activity of THP1‐Lucia ISG cells (Figure 4G; Figure S4F, Supporting Information). As A549 cells were not activated by 5 µM cisplatin, no synergistic effect was observed (Figure 4G). Together, these results demonstrate that the ENPP1 blockade of tumor cells and tumor‐derived exosomes in the tumor microenvironment may be a promising antitumor strategy by innate immune regulation. The combination of chemotherapy drugs and ENPP1 inhibitors may have special advantages for tumor treatment. In summary, our work reveals a new mechanism by which tumor cells can secrete exosomes that carry ENPP1 to effectively hydrolyze 2′3′‐cGAMP and LL‐37‐2′3′‐cGAMP in the tumor microenvironment and, therefore, inhibit the cGAS‐STING pathway in immune cells (Figure 4H).

### 2.5 Tumor Exosomal ENPP1 is Associated with Immune Suppression in Human Cancer

**Figure 5:**
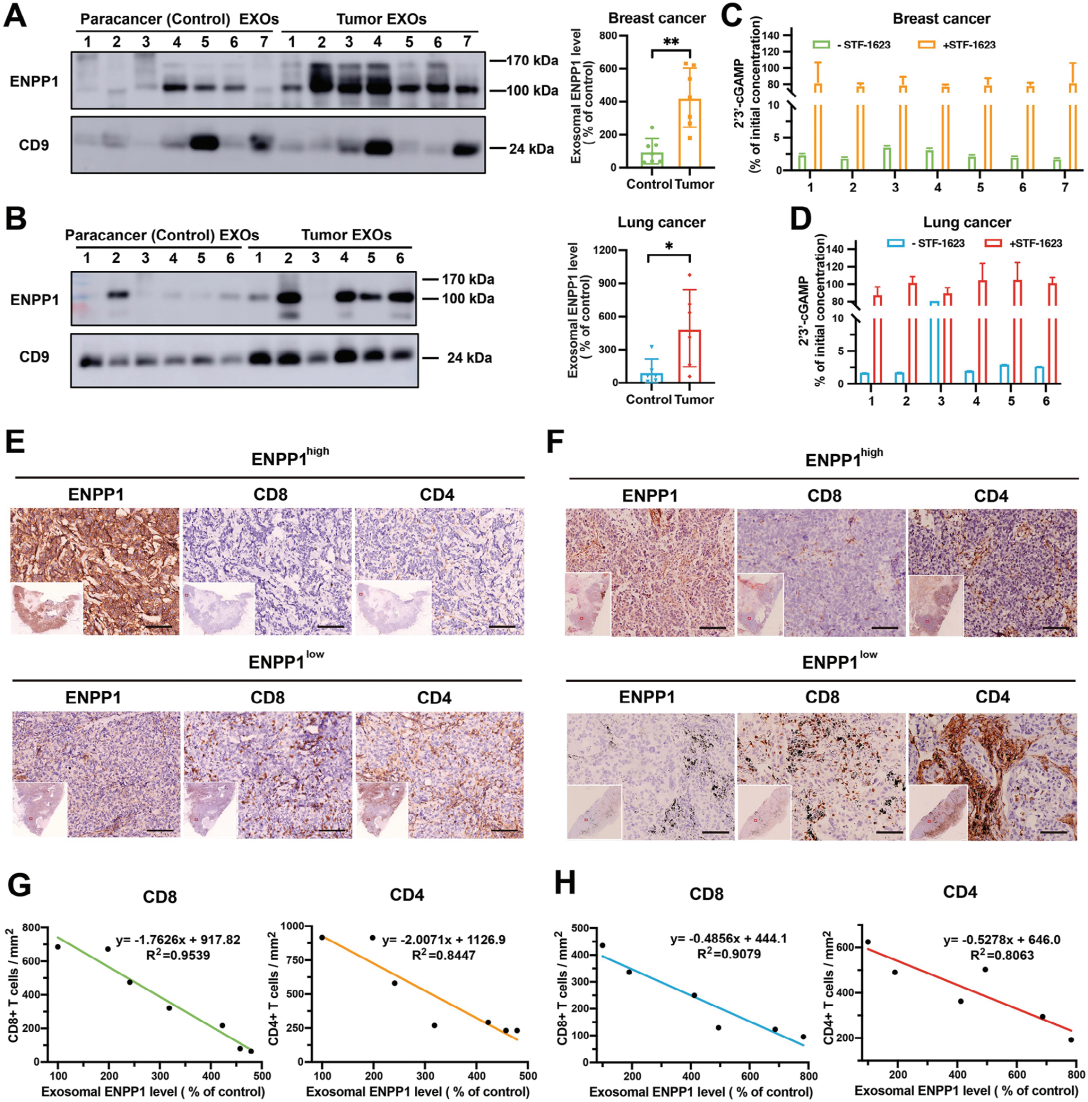
Tumor exosomal ENPP1 is associated with immune suppression in human cancer. A) Western blot analysis of ENPP1 in the exosomes purified from the paracancer tissue and tumor tissue of seven breast cancer patients (left). Quantification of the exosomal ENPP1 level determined by western blot analysis by Image J software (right). B) Western blot analysis of ENPP1 in the exosomes purified from the paracancer tissue and tumor tissue of six lung cancer patients (left). Quantification of the exosomal ENPP1 level determined by western blot analysis by Image J software (right). All p values were determined by unpaired two‐tailed Student's *t* test. NS, *p* > 0.05, *p < 0.05, **p < 0.01, ***p < 0.001, ****p < 0.0001. C,D) The percentage of residual 2′3′‐cGAMP to initial 2′3′‐cGAMP at 24 h of exosomes from breast tumor tissue (C) or lung tumor tissue (D) and 2′3′‐cGAMP co‐incubation in the absence or presence of STF‐1623 (300 nM). Data are presented as mean ± SD (*n* = 2 independent experiments). E,F) Representative images of human breast cancers (E) and lung cancers (F) stained using anti‐ENPP1, anti‐CD8 or anti‐CD4 antibodies. Scale bar, 100 µm. Nano Zoomer S60 scanner was used for scanning the stained slides. G,H) Correlation analysis of human breast cancers‐derived exosomal ENPP1 expression level (G) or lung cancers‐derived exosomal ENPP1 expression level (H) and the number of CD8+ T cells and CD4+ T cells infiltration per unit area. The exosomal ENPP1 level is the relative intensity of the lowest WB intensity of exosomal ENPP1. The number of CD8+ T or CD4+ T cells per unit area was counted in five random visual fields.

At the end of our project, we sought to explore the role of tumor exosomal ENPP1 in human cancers by analyzing various tumor tissues. We first surveyed ENPP1 protein expression in primary skin cutaneous melanoma (SKCM), ductal carcinoma of the breast (DCIS), non‐small cell lung cancer (NSCLC), colorectal cancer (COAD), and glioma by immunohistochemical (IHC) staining experiments. Consistent with reports,^[^ ^16^ ^]^ we observed three different expression patterns of ENPP1 in these tumors, which comprised negative, tumor cell dominant, and stroma dominant (Figure S5A, Supporting Information). This suggests that ENPP1 may exist in other forms in the tumor microenvironment, in addition to its membrane‐bound form on tumor cells. Breast and lung cancers were studied further, due to the relative ease of obtaining clinical samples. To determine the expression of ENPP1 in exosomes in the tumor microenvironment, exosomes were extracted from not only fresh tissues of breast and lung cancers, but also correspinding paracancer tissues. We observed little difference in the numbers and total protein levels of exosomes isolated from these tumor and paracancer tissues (Figure S5B,C, Supporting Information). In addition, western blot results revealed that the level of exsomal ENPP1 was higher in tumor tissues than in paracancer tissues (**Figure** **5**A,B). Moreover, our data reveal that all ENPP1‐carrying exosomes extracted from tumor tissues can effectively degrade 2′3′‐cGAMP (Figure 5C,D), which suggests that tumor‐derived exosomes are likely to hydrolyze 2′3′‐cGAMP in the tumor microenvironment by surface ENPP1 and dampen immune detection. We next correlated tumor exosomal ENPP1 levels with CD8+ T cells and CD4+ T cells density across breast cancers and lung cancers, and found that total ENPP1 of breast cancers or lung cancers tissue inhibited the immune infiltration of CD8+ T cells and CD4+ T cells (Figure 5E,F). Indeed, analysis from the TCGA database and early reports reveal that ENPP1 inhibits the infiltration of related immune cells (Figure S5D, Supporting Information),^[^ ^16^ ^]^ and high ENPP1 expression is associated with the poor prognosis of many cancers and the reduced overall survival of some cancer patients (Figure S5E, Supporting Information). Strikingly, the Western blot expression intensity of tumor exosomal ENPP1 also shown an inverse correlation with CD8+ T cells and CD4+ T cells infiltration, respectively (Figure 5G,H). Our studies suggest that breast and lung cancer release ENPP1‐positive exosomes into the tumor microenvironment to counter the anti‐tumor immunity. Our results also raise the possibility that disrupting hydrolytic activity of tumor exosomal ENPP1 in ENPP1 blocking therapy is a previously unrecognized mechanism in cGAS‐STING pathway. Collectively, these findings complement the results of our previous in vitro experiments and further demonstrate the important role of tumor exosomal ENPP1 in cancer.

## 3 Discussion

In the study described herein, we reveal a new mechanism by which tumor exosomal ENPP1 can hydrolyze 2′3′‐cGAMP effectively to inhibit cGAS‐STING pathway activation in immune cells. As an endogenous second messenger, 2′3′‐cGAMP plays an important role in innate immune response.^[^ ^1^, ^2^, ^3^, ^4^ ^]^ Although previous studies have shown that the ENPP1 protein on the cell surface membranes of tumors can impair immune response by hydrolyzing 2′3′‐cGAMP,^[^ ^5^, ^17^, ^18^ ^]^ the mechanisms affecting STING activation dependent on other forms of ENPP1 proteins had remained poorly understood. We found not only that exosomes derived from various tumor cells express ENPP1 protein, but also that ENPP1 is abundantly enriched on exosomes. Indeed, in all the cell types that we have tested, we observed that various tumor‐derived exosomes can hydrolyze 2′3′‐cGAMP effectively, which suggests that (1) the hydrolysis of 2′3′‐cGAMP by tumor exosomal ENPP1 is not a cancer‐specific phenomenon and (2) all tumor‐derived exosomes carrying ENPP1 may have the function of hydrolyzing 2′3′‐cGAMP. These exosomes exhibit dual regulation functions that not only inhibit cGAS‐STING signaling activation of immune cells due to the ENPP1 clearance mechanism, but also assist 2′3′‐cGAMP in enhancing the cGAS‐STING signaling pathway of immune cells. We hypothesize that exosomes may contain new transporters of 2′3′‐cGAMP. Nevertheless, ENPP1 inhibition of exosomes enhances the 2′3′‐cGAMP‐mediated STING signaling response of immune cells. These findings highlight the key function of tumor‐derived exosomes in the cGAS‐STING signaling pathway.

As an immuno‐transmitter, 2′3′‐cGAMP can be transported through a channel or by a transporter.^[^ ^6^, ^7^, ^8^, ^9^, ^10^, ^11^, ^12^, ^13^, ^14^, ^15^ ^]^ Recent studies have shown that LL‐37, as an endogenous antimicrobial peptide, is an important intercellular transporter of 2′3′‐cGAMP to significantly activate cGAMP‐induced immune response.^[^ ^15^ ^]^ We found that exosomes derived from various tumor cell types can impair 2′3′‐cGAMP‐mediated STING responses by hydrolyzing LL‐37‐2′3′‐cGAMP. These exosomes did not assist LL‐37‐2′3′‐cGAMP to enhance the cGAS‐STING signaling, which may be due to the inability of exosomes to transport LL‐37‐2′3′‐cGAMP. Exosomes are secreted actively by cells and widely exist in a variety of body fluids. In vivo, tumor cells probably secrete ENPP1‐positive exosomes to accelerate the hydrolysis of extracellular 2′3′‐cGAMP and LL‐37‐2′3′‐cGAMP, which promotes tumor immune evasion. Indeed, the ability of tumor exosomal ENPP1 to hydrolyze other transporter‐2′3′‐cGAMP is not excluded, and it further enhances the function of tumor‐derived exosomes in innate immune regulation.

Immune checkpoint blockade (ICB) therapy is largely ineffective against immune “cold” tumors that lack tumor‐infiltrating lymphocytes.^[^ ^40^ ^]^ Although STING is a novel immunotherapeutic target for converting “cold” tumors into “hot” tumors,^[^ ^41^ ^]^ STING agonists are non‐targeted and can activate STING in both cancer cells and the host, which promotes potential side effects.^[^ ^42^ ^]^ Meanwhile, the ENPP1 protein is selectively upregulated in chromosomally unstable tumors, and ENPP1 protein with cGAMP‐hydrolyzing activity is expressed on the membrane surface, which makes it safer and more convenient to regulate antitumor immunity by blocking ENPP1.^[^ ^43^ ^]^ At present, pharmaceutical companies have developed inhibitors that target ENPP1.^[^ ^19^, ^20^ ^]^ Our results suggest that endogenous 2′3′‐cGAMP produced by cells could be exported to the extracellular space and be hydrolyzed by tumor exosomal ENPP1. This would influence the interaction between paracrine tumor cells and bystander immune cells by inhibiting cGAS‐STING signaling pathway. In this context, the ENPP1 blockade of tumor‐derived exosomes could hold importance in immunotherapy. In addition, exosomes can assist endogenous 2′3′‐cGAMP to enhance the cGAS‐STING signaling in bystander cells. We show that a combination of chemotherapy drugs and ENPP1 inhibitors yields synergistic efficacy to enhance the STING signaling that is induced by 2′3′‐cGAMP. Nevertheless, the specific role of tumor‐derived exosomes in combination therapy requires further study. In summary, our study identifies a new molecular mechanism for tumor immune evasion by tumor exosomal ENPP1. The work also provides a possible approach for combined immunotherapy that features chemotherapeutic agents, ENPP1 inhibitors, and exosomes.

By examining the innate immune regulation function of tumor‐derived exosomes in vitro, we further elucidated the role of ENPP1 in a broader clinical setting. Consistent with previous reports,^[^ ^16^ ^]^ we observed a positive stroma expression pattern of ENPP1 in a variety of human cancer tissues, which suggests that tumor immune evasion is not only regulated by the tumor cells themselves, but also related to mediators in the tumor microenvironment, such as exosomes. Indeed, we observed high expression of ENPP1 in exosomes from breast and lung cancer tissue samples, and tumor exosomal ENPP1 inhibited the immune infiltration of CD8^+^ T cells and CD4^+^ T cells. This suggests that tumors secrete ENPP1‐positive exosomes to promote immune evasion by hydrolysis of 2′3′‐cGAMP and transporter‐2′3′‐cGAMP in the tumor microenvironment. In fact, exosomes have long half‐life and high stability in vivo.^[^ ^44^ ^]^ Relative to ENPP1 of tumor cells, tumor‐derived exosomes carrying ENPP1 can not only function in the microenvironment of primary tumors to assist tumor immune escape, but may also be transported remotely to participate in systemic immune regulation. Collectively, tumor‐derived exosomes, as important mediators of cell‐cell communication, should be studied for their mediated innate immune regulation in the tumor microenvironment.

## 4 Experimental Section

### Reagents, Antibodies, and Cells

Purchased from the indicated manufacturers were: ENPP1‐IN‐1 (HY‐129490, MCE), Gabexate mesylate (HY‐B0385, MCE), ADP (S9368, Selleck), 2′3′‐cGAMP (B8362, Apexbio), Cy3‐NHS ester (FY37537, Shanghai Feiyubio Ltd.), HT‐DNA (D6898, Sigma), STING‐IN‐2 (HY‐138682, MCE), EZ Trans (AC04L091, Shanghai Life iLab Bio Technology Co., Ltd.), GW4869 (HY‐19363, MCE), Quanti‐Luc (rep‐qlc1, InvivoGen), LL‐37 peptide (Shanghai GL Biochem Ltd.), TAT peptide (Shanghai GL Biochem Ltd.), STF‐1623 (Nantong Hi‐future Biotechnology Co., Ltd.), and 2′3′‐cGAMP ELISA Kit (501 700, Cayman).

Anti‐human ENPP1 (ab223268), anti‐human CD9 (ab263019), anti‐human CD9 (ab254175), and anti‐mouse ENPP1 (ab217368) were purchased from Abcam. Anti‐human CD8 (GT2112) and anti‐human CD4 (GT2191) were purchased from Gene Tech. Anti‐Flag (AF519), anti‐Actin (AF5003), anti‐rabbit lgG (A0208), anti‐mouse lgG (A0216), anti‐rat lgG (A0192) and anti‐mouse lgG, Alexa Flour 647 (A0473) were purchased from Beyotime Biotechnology. Anti‐mouse CD9 (14‐0091‐82) was purchased from eBioscience. Anti‐human ENPP1, Alexa Flour 488 (FAB6136G) and anti‐sheep lgG, Alexa Flour 488 (IC016G) were purchased from R&D Systems. 5 nm Colloidal Gold‐anti‐rabbit lgG (G7277) was purchased from Sigma.

A549 (CCL‐185), A375 (CRL‐1619), SW480 (CCL‐228), HeLa (CCL‐2), MDA‐MB‐231 (HTB‐26), LLC‐1 (CRL‐1624), B16F10 (CRL‐6475), 4T1 (CRL‐2539) and HEK 293T (CRL‐3216) were obtained from ATCC. U251 (89 081 403) was purchased from ECACC. RAW‐Lucia ISG cells (rawl‐isg) were purchased from InvivoGen. CAL27, MC38, and THP‐1 cells were gifts from Prof. Gang Chen of Wuhan University. THP1‐Lucia ISG cells were gifted from Prof. Conggang Zhang from Tsinghua‐Peking Center for Life Sciences.

### Cell Culture and Plasmids

Human embryonic kidney (HEK) 293T, human tumor cell lines A549, A375, CAL27, SW480, HeLa, MDA‐MB‐231, and U251, and mouse tumor cell lines LLC‐1, B16F10, MC38, 4T1 were cultured in DMEM medium (InvitroGen), supplemented with 10% fetal bovine serum (FBS, Gibco) and 1% penicillin‐streptomycin (P/S, Gibco) in a humidified incubator (Thermo) of 5% CO_2_ and 95% air at 37 °C. THP‐1 cells were maintained in RPMI 1640 medium, supplemented with 10% FBS and 1% P/S in a 5% CO_2_ incubator at 37 °C. THP1‐Lucia ISG cells were maintained in RPMI 1640 medium, supplemented with 10% FBS, 1% P/S, 2 mM L‐glutamine, 25 mM HEPES, and 100 µg mL^−1^ Normocin (Invivogen) in a 5% CO_2_ incubator at 37 °C. To maintain the selection pressure, 100 µg mL^−1^ Zeocin (Invivogen) was added to the medium every other passage. RAW‐Lucia ISG cells were maintained in DMEM medium, supplemented with 10% FBS, 1% P/S, 4.5 g L^−1^ glucose, 2 mM L‐glutamine, and 100 µg mL^−1^ Normocin (Invivogen) in a 5% CO_2_ incubator at 37 °C. To maintain the selection pressure, 200 µg mL^−1^ Zeocin (Invivogen) was added to the medium every other passage. The THP1‐Lucia ISG cells were provided by the Conggang Zhang lab.

To establish stable overexpression cells, cDNAs encoding human ENPP1 were cloned into H102 pLenti‐CMV‐3FLAG‐PGK‐Puro lentiviral vectors. First, 6 µg lentiviral plasmid DNA or empty vector was cotransfected with 3 µg RRE, 1.8 µg VSVG, and 1.2 µg REV to a 10‐cm dish of HEK 293T cells and transfected into cell lines. After 48 h, the culture medium containing lentivirus was centrifuged and collected at 1000 rpm for 10 min at room temperature. The A375, A549, MDA‐MB‐231, and SW480 cells were infected by virus for 24 h. Subsequently, the infected cells were selected with puromycin (1 ng µL^−1^) and tested by immunoblotting.

To make stable A375 eGFP and A375 ENPP1‐eGFP cells (eGFP was inserted C‐terminal of human ENPP1), cDNAs encoding eGFP or human ENPP1 were cloned into a H102 pLenti‐CMV‐3FLAG‐PGK‐Puro lentiviral vector. HEK 293T cells in a 10‐cm dish were cotransfected with 6 µg lentiviral plasmid DNA, 3 µg RRE, 1.8 µg VSVG, and 1.2 µg REV plasmids. The lentivirus was collected and infected with A375 cells as described above. Subsequently, the infected cells were selected with puromycin (1 ng µL^−1^) and tested by fluorescence microscopy.

To make A375, MDA‐MB‐231, and A549 ENPP1 knockdown (KD) cells, short hairpin RNAs (shRNAs) sequences targeting human ENPP1 cloned into a PLKO.1‐PURO vector. 6 µg shRNA is transfected directly into the target cells. Subsequently, the cells were cultured for 48 h and tested by Western Blotting. Four distinct shRNA sequences were screened for ENPP1 target. Targeted shRNA sequences are listed in Table S1 (Supporting Information).

### Purification of Exosomes

Exosomes were isolated from the conditioned media of exosome‐depleted FBS by differential centrifugation as previously described.^[^ ^45^ ^]^ Briefly, cells were maintained in exosome‐depleted FBS medium for 48 h to obtain the conditioned medium. Then, the conditioned medium was centrifuged at 3000 g (Beckman Coulter, Allegra X‐14R) for 30 min at 4 °C to remove the cell debris and apoptotic cells, followed by 16 000 g (Beckman Coulter, Optima XPN‐100) for 1 h to remove relatively larger microvesicles. Finally, the supernatant was centrifuged at 120 000 g for 2 h at 4 °C to collect the exosomes, followed by washing with 0.1X DPBS and centrifuging at 120 000 g for another 2 h. The exosomes were characterized by TEM (Tokyo Hitachi‐7700), NanoSight NS300 (Malvern Instruments), and western blot.

All tumor tissue mentioned herein was obtained according to procedures approved by the Ninth People's Hospital (Shanghai, Ethics approval number: SH9H‐2023‐T199‐2). Tissue‐derived exosomes were obtained as previously described.^[^ ^46^ ^]^ First, the tissue was washed with pre‐cooled 0.1X DPBS and drained with sterile gauze. Then, the tissues were minced and digested in DMEM, supplemented with 0.5 mg mL^−1^ type IV collagenase (Sigma) and 0.1 mg mL^−1^ DNase I (Sigma), and incubated with 500 rpm at 37 °C for 1 h. Suspensions were filtrated with a 70 µm cell strainer and collected to extract exosomes. The liquid was centrifuged at 300 g for 10 min and 2000 g for 20 min at 4 °C to remove the apoptotic cells and cell debris, followed by 10 000 g (Beckman Coulter, Allegra X‐14R) for 45 min to remove collagenase granules and relatively larger microvesicles. The supernatant was further centrifuged at 120 000 g (Beckman Coulter, Optima XPN‐100) for 70 min to collect the exosomes and resuspended with 0.1X DPBS. The final exosome pellets were stored at −80 °C until use. These tumor tissues used for exosome extraction were partially cut and were fabricated into the formalin‐fixed and parrffin‐embedded (FFPE) tumor tissue blocks for subsequent Immunohistochemistry (IHC) experiments.

### Fabrication of Liposomes

Liposomes were prepared using a thin film hydration and extrusion method.^[^ ^47^ ^]^ A mixture of DOPC, DPPC, DOPS, and cholesterol in a molar ratio of 1:1:0.5:1 was dissolved in chloroform and formed into a thin film, using rotary evaporation. The lipid film then was hydrated with DPBS at 37 °C for 1 h, allowing the formation of vesicles. To achieve optimal size and uniformity, the hydration mixture underwent 21 cycles of extrusion, through a 100 nm membrane, using a Mini‐Extruder. The liposomes were stored at 4 °C and intended for use within 1 week to maintain their stability.

### Western Blotting

Western blotting was employed for the analysis of cell and exosomal proteins. The target cells and exosomes were lysed with radioimmunoprecipitation assay (RIPA) buffer. Then, the cellular and exosomal protein concentration was quantified by BCA method. The cell or exosome lysates (10 µg) were subjected to 10% SDS‐PAGE and further electro transferred to a nitrocellulose filter membrane (Millipore). Afterward, the membranes were blocked with 5% skimmed milk and incubated with indicated antibodies overnight at 4 °C, followed by incubation with horseradish peroxidase (HRP)‐conjugated polyclonal antibodies for 1 h at room temperature. The protein strips were imaged by a Gel Image System (Amersham, Typhoon 9410). CD9 was used as exosomes marker. Actin was used as a loading control.

### TEM and Immunogold Labeling

The morphology of A375 cells‐derived exosomes was characterizated by TEM. In summary, 5 µL of exosomes were dropped on the carbon‐coated copper grid for 20 min to vaporize the solvent in the oven. The exosomes were negatively stained with 1% phosphotungstic acid for 30 s, and the remaining solution was absorbed by filter paper. Finally, the exosomes were imaged using TEM (Tokyo, Hitachi‐7700). In addition, the exosomal surface ENPP1 protein was characterized by immunogold labeling. Exosomes first were fixed with 4% paraformaldehyde in PBS solution at room temperature for 10 min. Then 10 µL of exosomes were dropped on the copper grid with carbon‐coated film for 1 h. The grid was washed using PBS containing 50 mM glycine and blocked in blocking buffer (PBS containing 0.5% bovine serum albumin [BSA]) for 30 min. Then a primary antibody (rabbit monoclonal antibody to ENPP1) was dropped on the grid overnight at 4 °C. After cleaning away the primary antibody, anti‐gold nanoparticle‐tagged secondary antibodies (anti‐rabbit gold IgG) were added to the grid and incubated for 1 h in the dark. Subsequently, the grid was washed three times with DPBS and negatively stained with 1% phosphotungstic acid for 30 s. Finally, the grid was imaged using TEM.

### Flow Cytometry

Flow cytometry was employed for the analysis of exosomal surface ENPP1. First, the target exosomes were co‐incubated with latex aldehyde strain (Sigam) for 30 min at room temperature, followed by the addition of primary (anti‐human ENPP1 Alexa Flour 488) or secondary antibody (anti‐sheep lgG Alexa Flour 488) and shaking for 3 h at room temperature. Then, the mixture was centrifuged at 300 rpm for 5 min and cleaned with PBS three times for precipitation. The final exosome‐latex aldehyde strain pellets were resuspended in 0.1X DPBS and measured by flow cytometer (CytoFLEX, Beckman, USA). Data was analyzed using the FlowJo software (Version 10.6.2, Treestar Inc.).

### Determination of Tumor Exosomal ENPP1 Hydrolyzing 2′3′‐cGAMP

Exosomes derived from human tumor cells were placed in 1.5 mL tubes at a concentration of 0.5 mg mL^−1^ and incubated with 200 nM 2′3′‐cGAMP in PBS in the absence or presence of 1 µM ENPP1‐IN‐1 (ENPP1 inhibitor) at 37 °C. After varying reaction times (0, 0.5, 1, 1.5, 2, 4, 6, and 8 h), 1.2 µL suspensions were added to 70 µL PBS and heated at 95 °C for 5 min. Then, the 2′3′‐cGAMP of this mixture was detected by ELISA, according to the standard method (Cayman, Cat#: 501700). Gabexate mesylate (GM) was used as a control against ENPP1‐IN‐1, following the the same experimental procedure. Exosomes derived from mouse tumor cells were used at a concentration of 0.5 mg mL^−1^ for the same experiments.

In addition, 0.5 mg mL^−1^ of exsomes derived from human tumor cells were incubated with 40 µg mL^−1^ LL‐37 and 200 nM 2′3′‐cGAMP in PBS in the absence or presence of 1 µM ENPP1‐IN‐1 (ENPP1 inhibitor) at 37 °C. After varying reaction times (0, 0.5, 1, 2, 4, 8 h), 1.2 µL suspensions were added to 70 µL PBS and heated at 95 °C for 5 min. TAT (40 µg mL^−1^) was used as a control of LL‐37, following the same experimental procedure. Subsequently, the 2′3′‐cGAMP of this mixture is analyzed by ELISA. Exosomes derived from mouse tumor cells (0.5 mg mL^−1^) were used for the same experiments.

### Luciferase Reporter Assay

Human THP1‐Lucia ISG and Murine RAW‐Lucia ISG were derived from the human THP1 and murine RAW264.7 cell lines, respectively, by stable integration of luciferase reporter gene, under the control of five Interferon (IFN)‐Stimulated Response Elements (ISRE), fused to an ISG54 minimal promoter.^[^ ^29^, ^31^, ^32^ ^]^ The ISRE luciferase activity of Lucia ISG cells was proportional to the signal intensity of the cGAS‐STING pathway. THP1‐Lucia ISG cells were seeded in a 96‐well plate at a density of 50000 cells per well, followed by induction with 100 ng mL^−1^ PMA for 24 h. The cells were incubated with 200 nM 2′3′‐cGAMP, 150 ng µL^−1^ liposomes, a combination of 200 nM 2′3′‐cGAMP and 150 ng µL^−1^ liposomes, a combination of 200 nM 2′3′‐cGAMP, 150 ng µL^−1^ liposomes, and 300 nM STF‐1623, a combination of 200 nM 2′3′‐cGAMP, 150 ng µL^−1^ liposomes, and 300 nM ADP, 150 ng µL^−1^ EXOs, a combination of 200 nM 2′3′‐cGAMP and 150 ng µL^−1^ EXOs, a combination of 200 nM 2′3′‐cGAMP, 150 ng µL^−1^ EXOs, and 300 nM STF‐1623, or a combination of 200 nM 2′3′‐cGAMP, 150 ng µL^−1^ EXOs, and 300 nM ADP in fresh FBS‐free medium for 24 h. The ISRE reporter activity was determined according to a standard protocol (Invivogen). In addition, the THP1‐Lucia ISG cells were stimulated with 200 nM 2′3′‐cGAMP and different concentrations of A375 EXOs (60, 120, 180 ng µL^−1^) in the absence or presence of STF‐1623 (300 nM) for 24 h, and characterized for ISRE luciferase activity. Moreover, exosomes derived from different tumor cells (A549, MDA‐MB‐231, and SW480) were used to stimulate the THP1‐Lucia ISG cells with 200 nM 2′3′‐cGAMP in the absence or presence of STF‐1623 (300 nM) for 24 h and characterized following the standard protocol (InvivoGen, Cat#: rep‐qlc). Furthermore, the THP1‐Lucia ISG cells were stimulated with 200 nM 2′3′‐cGAMP and different exosomes from ENPP1‐overexpressed cells (A375, A549, MDA‐MB‐231, and SW480 ENPP1‐OE EXOs) in the absence or presence of STF‐1623 (300 nM) for 24 h. The RAW‐Lucia ISG cells were stimulated with 2′3′‐cGAMP (500 nM), exosomes from LLC‐1, or B16F10 cells in the absence or presence of STF‐1623 (300 nM) for 24 h and characterized following the standard protocol.

To detect the ability of tumor exosomal ENPP1 to hydrolyze LL‐37‐2′3′‐cGAMP, THP1‐Lucia ISG cells were treated with a combination of 40 µg mL^−1^ TAT and 200 nM 2′3′‐cGAMP, a combination of 40 µg mL^−1^ TAT, 200 nM 2′3′‐cGAMP, and 150 ng µL^−1^ EXOs in the absence or presence of STF‐1623 (300 nM), a combination of 40 µg mL^−1^ LL‐37 and 200 nM 2′3′‐cGAMP, or a combination of 40 µg mL^−1^ LL‐37, 200 nM 2′3′‐cGAMP, and 150 ng µL^−1^ EXOs in the absence or presence of STF‐1623 (300 nM), in fresh FBS‐free medium for 24 h. In addition, the THP1‐Lucia ISG cells were treated with a combination of LL‐37 (40 µg mL^−1^) and 2′3′‐cGAMP (200 nM) and different concentrations of A375 exosomes (60, 120, 180 ng µL^−1^) in the absence or presence of STF‐1623 (300 nM) for 24 h and characterized, following the standard protocol. Moreover, THP1‐Lucia ISG cells were treated with a combination of LL‐37 (40 µg mL^−1^) and 2′3′‐cGAMP (200 nM), a combination of LL‐37 (40 µg mL^−1^), 2′3′‐cGAMP (200 nM), and EXOs/ENPP1‐OE EXOs from different tumor cells in the absence or presence of STF‐1623 (300 nM) for 24 h and analyzed as described above. The RAW‐Lucia ISG cells also were stimulated with a combination of 40 µg mL^−1^ LL‐37 and 500 nM 2′3′‐cGAMP, a combination of 40 µg mL^−1^ LL‐37, 500 nM 2′3′‐cGAMP, and 150 ng µL^−1^ EXOs from LLC‐1 or B16F10 cells in the absence or presence of STF‐1623 (300 nM) for 24 h and characterized following the standard protocol.

### Fluorescence Microscopy

A375 cells that stably express an engineered eGFP/ ENPP1‐eGFP gene were seeded on cell crawlers, at a density of 1000 cells/well for 24 h. Subsequently, the cells were fixed with 4% paraformaldehyde and stained with DAPI, followed by observation using a Leica TCS SP8 CARS with a 63X objective. In addition, exosomes derived from A375 eGFP/A375 ENPP1‐eGFP cells were purified by ultracentrifugation, as described above, and observed using a Leica TCS SP8 CARS with a 63X objective.

To further examine the interaction of tumor‐derived exosomes and LL‐37‐2′3′‐cGAMP in cells using fluorescence microscopy, LL‐37 first was modified with Cy3 molecules. In summary, to a solution of LL‐37 (2 mg mL^−1^) in PBS, Cy3‐NHS ester (0.8 mg mL^−1^) was added at 800 rpm for 2 h at 37 °C. LL‐37 and Cy3 bind to form Cy3‐LL‐37 through amino and NHS reactions. Then the Cy3‐LL‐37 was purified by salinization columns and further concentrated using ultrafiltration tubes (3 kDa).

Subsequently, THP‐1 cells were seeded on cell crawlers at a density of 1000 cells per well. After induction by 100 ng mL^−1^ PMA for 24 h, cells were treated with 40 µg mL^−1^ Cy3‐LL‐37, 200 nM 2′3′‐cGAMP, and 150 ng µL^−1^ A375 ENPP1‐eGFP EXOs for 24 h. To remove residual Cy3‐LL‐37‐2′3′‐cGAMP, the cells were washed with PBS and further fixed with 4% paraformaldehyde for 10 min. Fixed cells were permeated with 0.5% Triton X‐100 in PBS for 15 min at 4 °C. Following permeabilization, cells were blocked with 5% BSA, and then incubated with the appropriate primary antibody (anti‐CD9) for 1 h at 37 °C. Following primary incubation, cells were incubated with the appropriate secondary antibody for 1 h at 37 °C. All wash steps were with PBS. Finally, the cells were stained with DAPI and observed using a Leica TCS SP8 CARS with a 63X objective.

### Purification of ENPP1 Protein

To investigate the enzymatic cleavage of endogenous 2′3′‐cGAMP by ENPP1 protein, ENPP1 protein first was purified from A375 cells that stably express an engineered ENPP1 gene with flag tag. In summary, 15 mL of cell lysis solution (1X PBS containing 10% glycerin and 0.3% Triton X‐100) was added to ≈10^8^ cells to fully lyse the cells using ultrasonic cell disrupter system (Branson, S‐450D). Then, the cell lysate was centrifuged at 15 000 rpm for 10 min, and the supernatant was collected; 100 µL anti‐flag affinity gel was added to the supernatant overnight at 4 °C to specifically bind of flag label ENPP1 proteins. ENPP1 protein captured by the anti‐flag affinity gel was enriched further by affinity chromatography and eluted using 3X flag peptide through competitive binding of anti‐flag antibodies. The filtrate containing ENPP1 protein was concentrated by ultrafiltration tube (10 kDa) at 10 000 rpm for 5 min and quantified by the Bradford Protein Quantification Kit. The purified proteins were charactered further by staining with Coomassie bright blue as follows: The purified proteins (20 µg) were separated by 10% SDS‐PAGE gel and stained further with Coomassie bright blue for 10 min. The dyed gel was decolorized using decolorization buffer (1X PBS containing 45% methanol and 10% acetic acid). Simultaneously, western blotting was employed for the analysis of ENPP1 proteins following the previous procedure.

### Co‐Cultured Experiments

To assess the role of tumor exosomal ENPP1 in endogenous 2′3′‐cGAMP and the effect on the cGAS‐STING pathway in bystander cells, co‐cultured experiments were performed. First, 50 ng mL^−1^ HT‐DNA was transfected into WT THP‐1 cells in a T25 cell culture bottle. After 6 h, the cell suspension was centrifuged and collected at 800 rpm for 5 min at room temperature. The WT THP‐1 cells were (1) washed twice with 0.1X DPBS to remove HT‐DNA that had not been transfected into cells and (2) mixed with THP1‐Lucia ISG cells. The cell suspension was dispensed into 96‐well plates (WT THP‐1 50000 cells/well, THP1‐Lucia ISG 50 000 cells per well), and co‐cultured for up to 48 h by adding 0.4 ng µL^−1^ above the purified ENPP1 protein or 40 ng µL^−1^ above the purified A375 EXOs/A375 ENPP1‐OE EXOs in the absence or presence of STF‐1623 (300 nM), respectively. The ISRE reporter activity at varying times was detected following the standard protocol. This control experiment was performed using WT THP‐1 cells treated without HT‐DNA to co‐culture with THP1‐Lucia ISG cells.

Similarly, either 50 ng mL^−1^ HT‐DNA or 5 µM cisplatin each were added to a 10‐cm dish of various tumor cells (A375, MDA‐MB‐231, A549, ENPP1‐overexpressed A375) or ENPP1 knockdown tumor cells (A375, MDA‐MB‐231, A549 ENPP1‐KD) for 6 h. Subsequently, the cells were washed with DPBS and detached with 0.25% trypsin/EDTA, followed by centrifugation (800 rpm for 5 min). These tumor cells further co‐cultured further with THP1‐Lucia ISG cells in 96‐well plates for up to 48 h with or without STF‐1623 (300 nM) treatment. The ISRE reporter activity at various times was detected following the standard protocol. This control experiment was performed using tumor cells that were not stimulated by the HT‐DNA or cisplatin.

### Immunohistochemistry (IHC)

All the formalin‐fixed and parrffin‐embedded (FFPE) tumor tissue blocks mentioned herein were obtained according to procedures approved by the Ninth People's Hospital (Shanghai). In brief, the FFPE tissue blocks were sliced into thin section slides and placed in an oven at 60 °C for 30–60 min. IHC was performed on tumor section slides using a Dako Autostainer Link 48 platform. The section slides were further dewaxed using EZ prep for 4 min at 72 °C. The sections immersed into Cell Conditioning Solution (CC1) were heated at 95 °C for 64 min for antigen retrieval. Cooled section slides were incubated with 3–5% H_2_O_2_ for 10 min at 37 °C to block endogenous oxidase. Subsequently, slides were incubated with primary antibody in blocking buffer for 20 min at 37 °C, followed by two washes. Sections then were incubated with HRP‐linked antibody for 8 min, followed by washing and visualization with DAB for DCIS, NSCLC, COAD, and glioma tissues. In addition, AEC chromogen was employed for visualization of SKCM tissue. Finally, the sections were incubated with Hematoxylin for 10 min and sealed. An Olympus BX41 microscope was used for scanning the stained slides.

### Synthetic Methods of STF‐1623

Sodium hydride (0.19 g, 4.74 mmol) was added to a stirred solution of tetramethyl methylenediphosphonate (1.0 g, 4.31 mmol) in toluene (10 mL) at 0 °C under nitrogen gas for 15 min. Then, 1 (0.876 g, 4.31 mmol) was added, and the mixture was stirred at room temperature for 13 h. Water was added and further extracted with 2×50 mL ethyl acetate The organic phase was separated, washed with brine, dried with Na_2_SO_4_, and concentrated and puried by flash to yield 3 (1.1 g, 82% yield), as a yellow oil. Pd/C (0.2 g) was added to a solution of 3 (0.9 g, 2.89 mmol) in ethanol (10 mL) at 0 °C under hydrogen gas for 24 h. The mixture then was quenched and puried by silica gel column chromatography to yield 4 (0.43 g, 68% yield), as a yellow oil. To a solution of 4 (0.43 g) in ethanol (10 mL), 5 (0.397 g) was added at 0 °C under nitrogen gas for 15 min. Then ethyldiisopropylamine (0.66 mL) was added to the stirred mixture, maintaining the temperature at 90 °C for 3 h. Then water was added and extracted with 2×50 mL ethyl acetate. Combined organics were washed with brine, dried with Na_2_SO_4_, and concentrated and puried by silica gel column chromatography to yield 6 (0.3 g, 38% yield), as a white solid. To a solution of 6 (0.1 g) in trichloromethane (5.0 mL), bromotrimethylsilane (0.337 g) was added at 0 °C under nitrogen gas. After stirring at 0 °C for 2 h, the mixture was extracted with ethyl acetate. The organic phase was separated, washed with brine, dried with Na_2_SO_4_, and concentrated and puried by silica gel column chromatography to yield STF‐1623 (30 mg), as a white solid.

1H NMR was performed on Bruker Avance Neo 600 spectrometers. NMR data was analyzed by MestReNova (version 6.1.0). NMR of STF‐1623 is shown in Figure S2D (Supporting Information).

## Conflict of Interest

The authors declare no conflict of interest.

## Author Contributions

Y.A., C.Y., T.J. performed conceptualization. J.F., Y.G., Q.F., J.C., Z.H., and W.S. performed data curation. J.F., Y.G., Q.F., J.C., W.S. performed formal analysis. Y.A., L.W., C.Y., T.J. performed funding acquisition. Y.A., J.Z. performed investigation. Y.A., Z.H., Q.L. performed methodology. L.W., C.Y., T.J. performed project administration. J.Z., Q.X., Q.L. accumulated the resources. J.F. acquired the software. C.Y., T.J. performed supervision. Q.X., J.C. performed validation. Y.G., Q.F. performed visualization. Y.A. and L.W. performed writing of original draft. L.W., C.Y., and T.J. performed writing, reviewing & editing.

## Supporting information

Supporting Information

## Data Availability

The data that support the findings of this study are available from the corresponding author upon reasonable request.
