# Supplementary material for: Tumor Exosomal ENPP1 Hydrolyzes cGAMP to Inhibit cGAS‐STING Signaling
Source: Adv Sci (Weinh). 2024 Mar 18;11(20):2308131. doi: 10.1002/advs.202308131 (PMC11132070; doi:10.1002/advs.202308131)
Supplement: Supplementary file 1 — Supporting Information [file ADVS-11-2308131-s001.pdf]

ADVANCED SCIENCE
Open Access

Supporting Information

for *Adv. Sci.*, DOI 10.1002/advs.202308131

# Tumor Exosomal ENPP1 Hydrolyzes cGAMP to Inhibit cGAS-STING Signaling

*Yu An, Jinchao Zhu, Qihui Xie, Jianzhou Feng, Yanli Gong, Qian Fan, Jiao Cao, Zhi Huang, Weixiong Shi, Qingyuan Lin, Lingling Wu*, Chaoyong Yang* and Tianhai Ji**

# Supplementary Information

## Tumor exosomal ENPP1 hydrolyzes cGAMP to inhibit cGAS-STING signaling

*Yu An[1], Jinchao Zhu[1], Qihui Xie[4], Jianzhou Feng[2], Yanli Gong[2], Qian Fan[2], Jiao Cao[2], Zhi Huang[2], Weixiong Shi[2], Qingyuan Lin[1], Lingling Wu[2]*, Chaoyong Yang[2,3]*, Tianhai Ji[1]**

[1] Department of Pathology, Shanghai Ninth People's Hospital, Shanghai Jiao Tong University School of Medicine, Shanghai 200011, P. R. China

[2] Institute of Molecular Medicine, Renji Hospital, Shanghai Jiao Tong University School of Medicine, Shanghai 200127, P. R. China

[3] The MOE Key Laboratory of Spectrochemical Analysis and Instrumentation, State Key Laboratory of Physical Chemistry of Solid Surfaces, Department of Chemical Biology, College of Chemistry and Chemical Engineering, Xiamen University, Xiamen 361005, P. R. China

[4] State Key Laboratory of Oral & Maxillofacial Reconstruction and Regeneration, Key Laboratory of Oral Biomedicine Ministry of Education, Hubei Key Laboratory of Stomatology, School & Hospital of Stomatology, Wuhan University, Wuhan 430070, P. R. China

*Corresponding Author: Tianhai Ji, **Email:** skysea_ji@sina.com; Chaoyong Yang, **Email:** cyyang@xmu.edu.cn; Lingling Wu, **Email:** llwu@shsmu.edu.cn.

**Table of Contents**

**Figure S1** Tumor cells-derived exosomes express ENPP1 and hydrolyze 2’3’-cGAMP/LL-37-2’3’-cGAMP........................................................................................................... 3

**Figure S2** Tumor exosomal ENPP1 inhibits cGAS-STING signaling by hydrolyzing 2’3’-cGAMP.............................................................................................................................4

**Figure S3** Tumor exosomal ENPP1 inhibits cGAS-STING signaling by hydrolyzing LL-37-2’3’-cGAMP........................................................................................................... 6

**Figure S4** Tumor exosomal ENPP1 hydrolyzes endogenous 2’3’-cGAMP produced by cells and inhibits cGAS-STING signaling in bystander cells...........................................................7

**Figure S5** Tumor tissue and tumor tissue-derived exosomes express ENPP1..........................8

**Table. S1** shRNA guide sequence.........................................................................................10

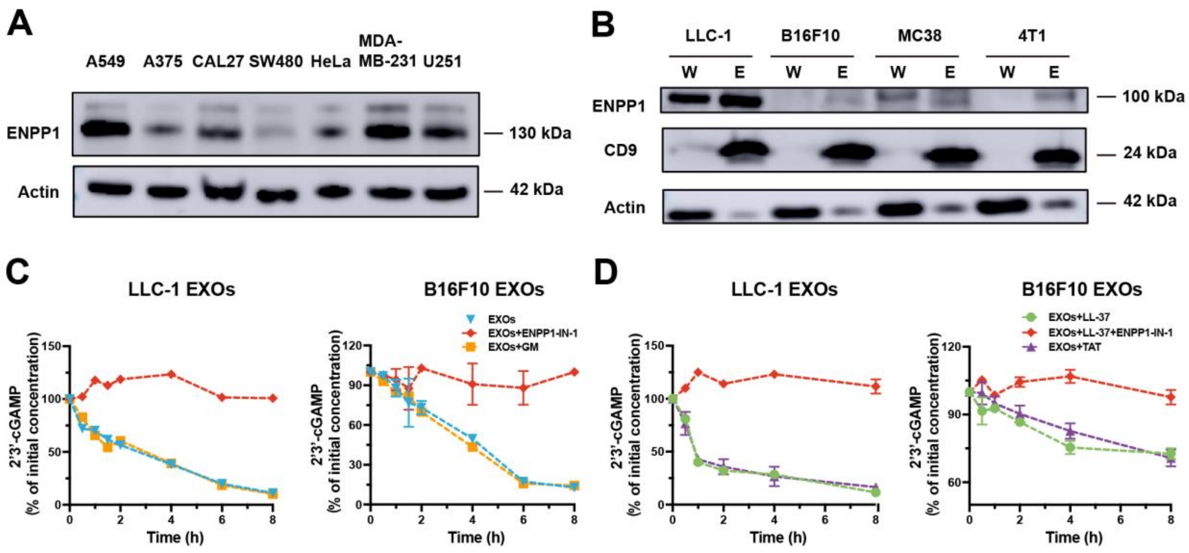


**Figure S1. Tumor cells-derived exosomes express ENPP1 and hydrolyze 2’3’-cGAMP/LL-37-2’3’-cGAMP.** (**A**) Western blot analysis of ENPP1 in whole cell lysate from different human tumor cell lines. All lanes were loaded with the same amount of total protein. (**B**) Western blot analysis of ENPP1 in whole cell lysate (W) and exosomes (E) from different mouse tumor cell lines. All lanes were loaded with the same amount of total protein. (**C**) The percentage of residual 2’3’-cGAMP to initial 2’3’-cGAMP at different times of exosomes (0.5 mg/mL) and 2’3’-cGAMP (200 nM) co-incubation. Exosomes from LLC-1 and B16F10 cells, with or without ENPP1-IN-1/GM treatment. (**D**) The percentage of residual 2’3’-cGAMP to initial 2’3’-cGAMP at different times of exosomes (0.5 mg/mL), LL-37 (40 μg/mL) and 2’3’-cGAMP (200 nM) co-incubation. TAT (40 μg/mL) was selected as the negative control of LL-37. Data are presented as mean ± SD (n=2 independent experiments).

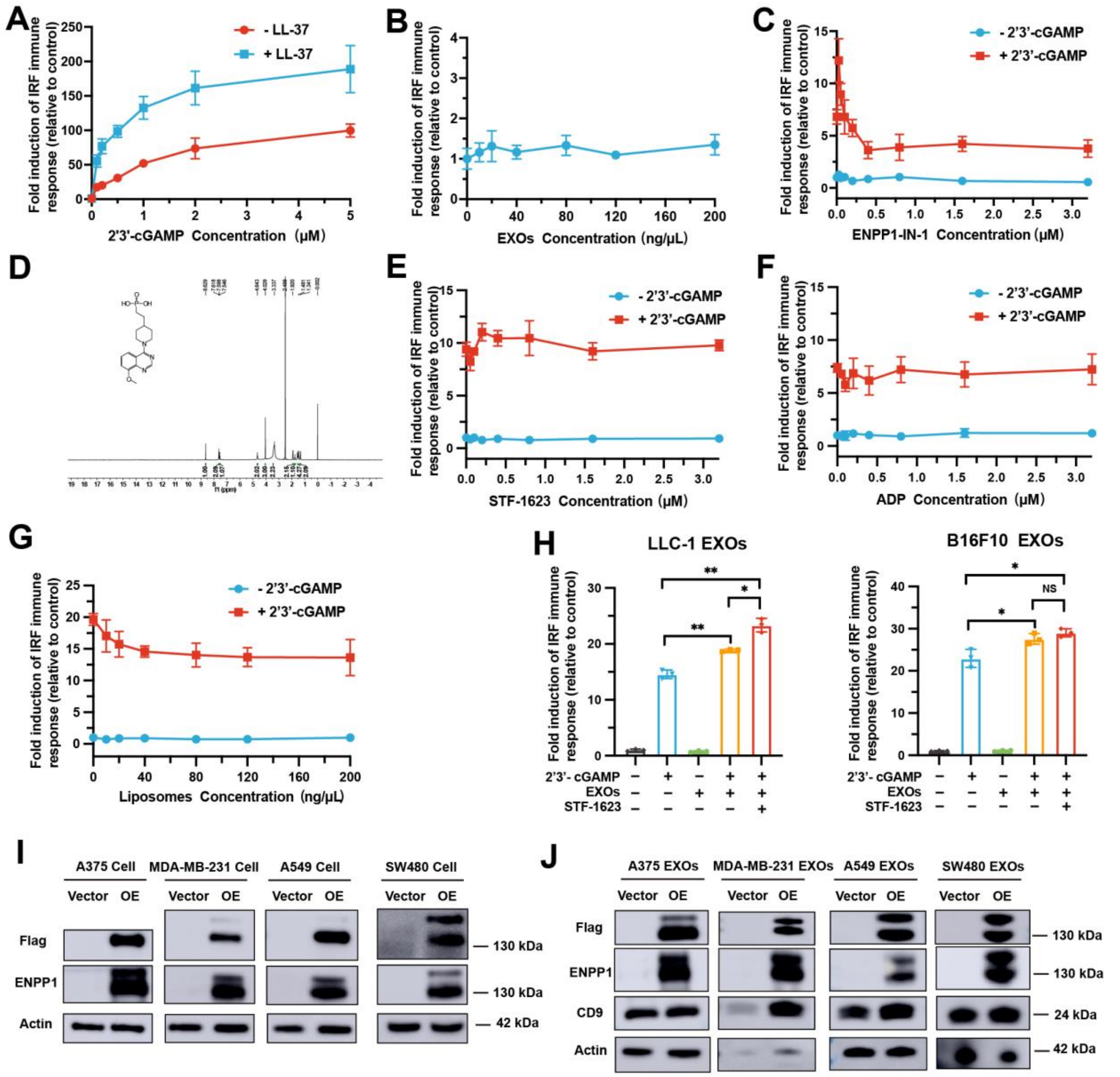


**Figure S2. Tumor exosomal ENPP1 inhibits cGAS-STING signaling by hydrolyzing 2'3'-cGAMP.** (**A**) ISRE reporter activity analysis of THP1-Lucia ISG cells stimulated with different concentrations (0.1, 0.2, 0.5, 1.0, 2.0, 5.0 μM) of 2'3'-cGAMP in the absence or presence of LL-37 (40 μg/mL) for 24 hours. (**B**) ISRE reporter activity analysis of THP1-Lucia ISG cells treated with increasing concentrations (10, 20, 40, 80, 120, 200 ng/μL) of A375 EXOs for 24 hours. (**C**) ISRE reporter activity analysis of THP1-Lucia ISG cells treated with different concentrations (0.05, 0.1, 0.2, 0.4, 0.8, 1.6, 3.2 μM) of ENPP1-IN-1. (**D**) Structure and $^{1}$H NMR of the ENPP1 inhibitor STF-1623 in $CDCl_3$ (500 M Hz, 298K). (**E, F**) ISRE reporter activity analysis of THP1-Lucia ISG cells treated with different concentrations of STF-1623 (0.05, 0.1, 0.2, 0.4, 0.8, 1.6, 3.2 μM) (**E**) or ADP (0.05, 0.1, 0.2, 0.4, 0.8, 1.6, 3.2 μM) (**F**) in the absence

or presence of 2’3’-cGAMP (200 nM) for 24 hours. (**G**) ISRE reporter activity analysis of THP1-Lucia ISG cells stimulated with increasing concentrations (10, 20, 40, 80, 120, 200 ng/μL) of liposomes with or without 2’3’-cGAMP (200 nM) for 24 hours. (**H**) ISRE reporter activity analysis of THP1-Lucia ISG cells treated with 2’3’-cGAMP (500 nM), LLC-1 EXOs (150 ng/μL), a combination of 2’3’-cGAMP and LLC-1 EXOs, or a combination of 2’3’-cGAMP, LLC-1 EXOs and STF-1623 (300 nM) (left) for 24 hours. Similar experiment was performed using B16F10 EXOs (right). Data are presented as mean ± SD of three independent experiments (Figure S2A-C, E-H). All p values were determined by ANOVA. NS, $p > 0.05$, *$p < 0.05$, **$p < 0.01$, ***$p < 0.001$, ****$p < 0.0001$. (**I**) Western blot analysis of ENPP1-overexpressed (ENPP1 OE) cell lysates from different human tumor cells. All lanes were loaded with the same amount of total protein. (**J**) Western blot analysis of exosomes from ENPP1-overexpressed tumor cells. The same amount of protein was loaded in each lane.

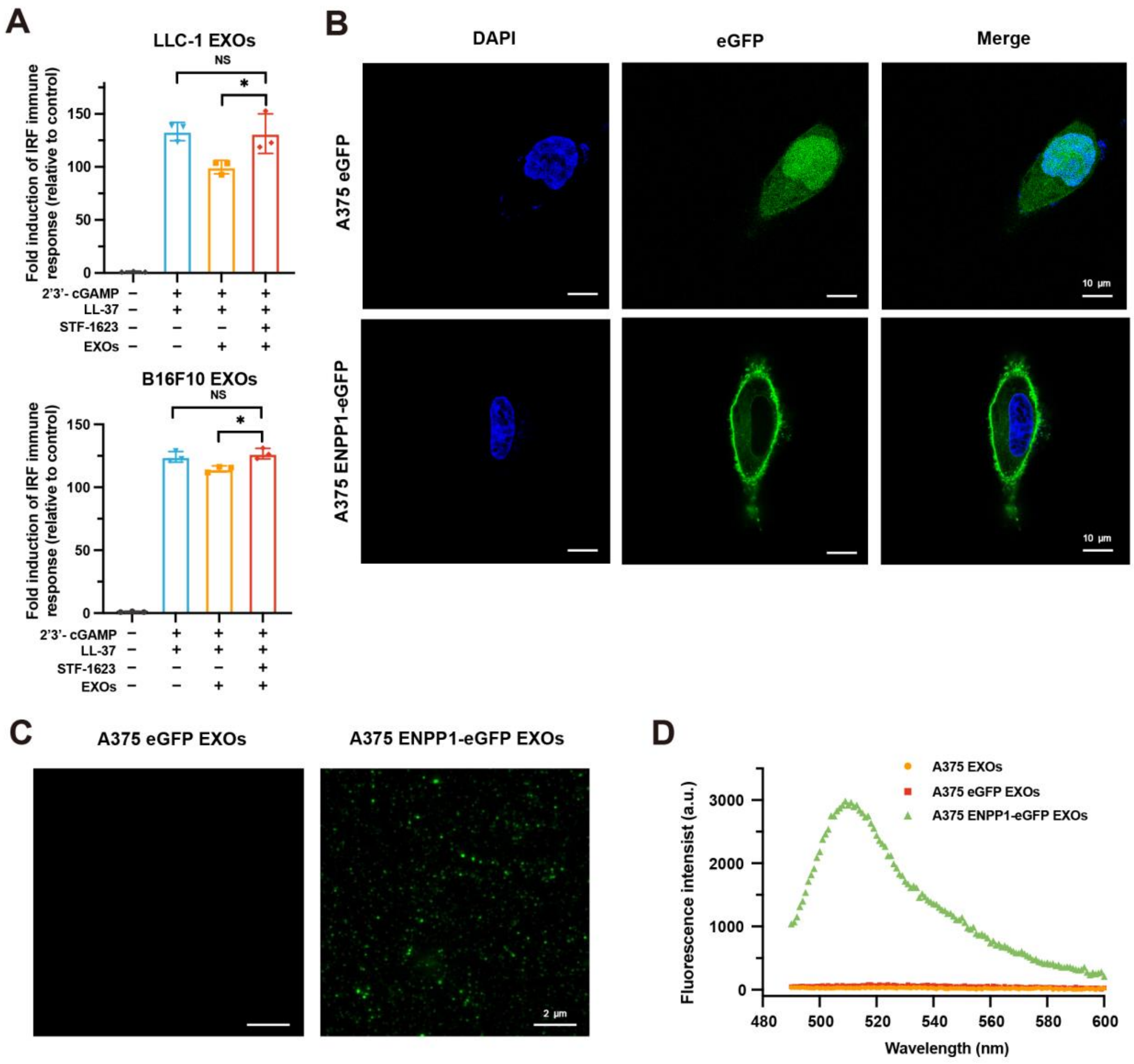


**Figure S3. Tumor exosomal ENPP1 inhibits cGAS-STING signaling by hydrolyzing LL-37-2'3'-cGAMP.** (**A**) ISRE reporter activity analysis of THP1-Lucia ISG cells treated with LL-37 (40 μg/mL)-2'3'-cGAMP (500 nM), a combination of LL-37-2'3'-cGAMP and LLC-1 EXOs (150 ng/μL) (top) in the absence or presence of STF-1623 (300 nM) for 24 hours. Similar experiments were performed using B16F10 EXOs (bottom). Data are presented as mean ± SD (n=3 independent experiments). All p values were determined by ANOVA. NS, $p > 0.05$, *$p < 0.05$, **$p < 0.01$, ***$p < 0.001$, ****$p < 0.0001$. (**B**) Fluorescence image of eGFP/ENPP1-eGFP overexpressed A375 cells with the Leica TCS SP8 CARS using a 63X objective. (**C**) Fluorescence image of A375 eGFP/ENPP1-eGFP EXOs with the Leica TCS SP8 CARS using a 63X objective. (**D**) Fluorescence intensity determination of A375 eGFP/ENPP1-eGFP EXOs using Synergy H1.

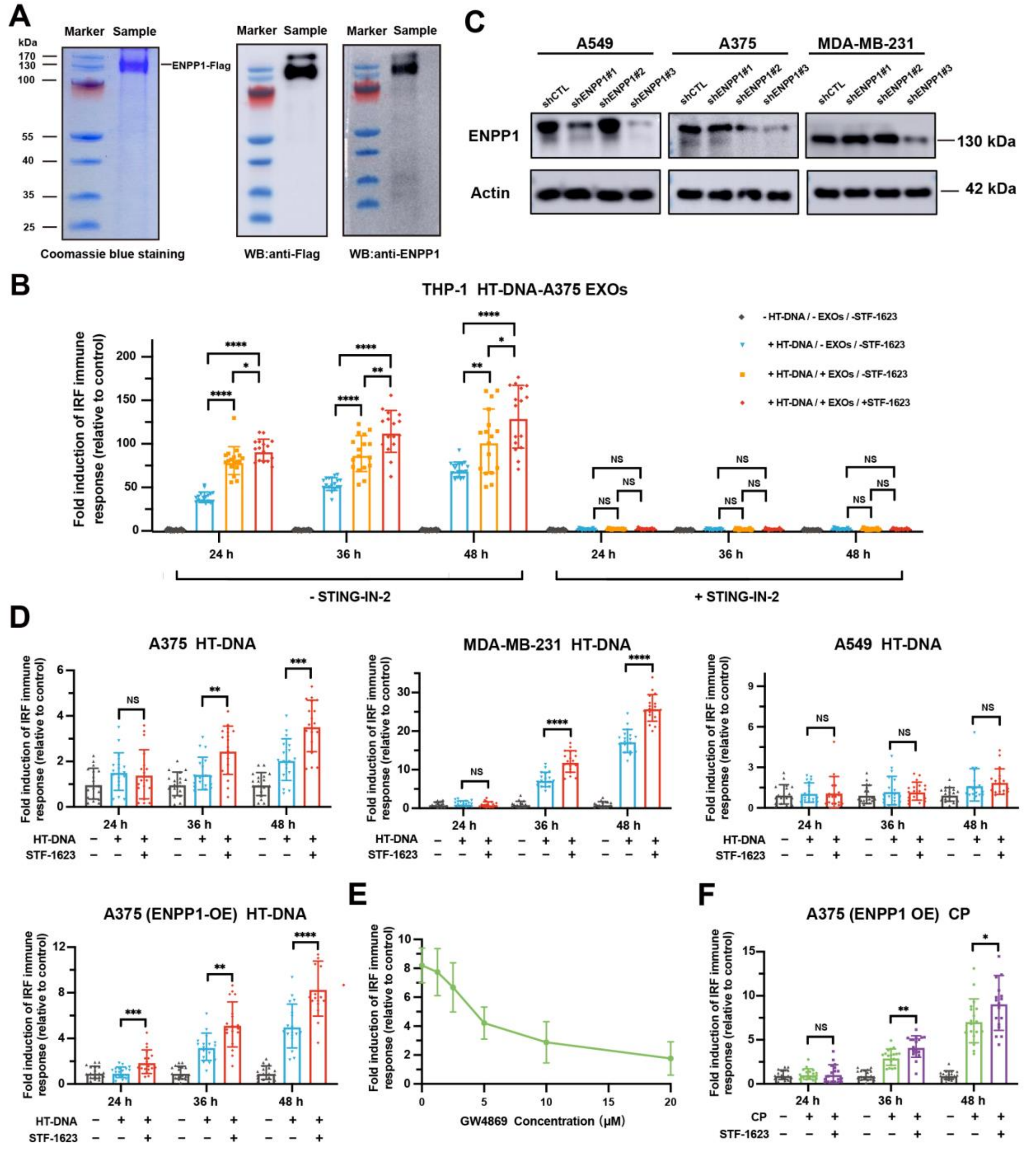


**Figure S4. Tumor exosomal ENPP1 hydrolyzes endogenous 2’3’-cGAMP produced by cells and inhibits cGAS-STING signaling in bystander cells.** (**A**) Coomassie blue staining analysis of purified ENPP1 protein from ENPP1-overexpressed A375 cells (left). Western blots analysis of purified ENPP1 protein from ENPP1-overexpressed A375 cells using anti-Flag (middle) and anti-ENPP1 antibodies (right). (**B**) ISRE reporter activity analysis of THP1-Lucia ISG cells co-cultured with HT-DNA (50 ng/mL)-treated WT THP-1 cells for up to 48 hours with or without STING-IN-2 (1 μM) (n=18 independent experiments). (**C**) Western blot analysis of A549, A375 and MDA-MB-231 cells examined for the expression of ENPP1 with or without

ENPP1 knockdown. A549, A375 and MDA-MB-231 cells were treated with shRNA against ENPP1 (shENPP1) or the scrambled control shRNA (shCTL), respectively. (**D**) ISRE reporter activity analysis of THP1-Lucia ISG cells co-cultured with HT-DNA (50 ng/mL)-treated different human tumor cells (A375, MDA-MB-231, A549, and ENPP1-overexpressed A375 cells) with or without STF-1623 (300 nM) for up to 48 hours (n=18 independent experiments). (**E**) ISRE reporter activity analysis of THP1-Lucia ISG cells stimulated with increasing concentrations (0, 1.25, 2.5, 5, 10, 20 μM) of GW4869 for 24 hours (n=6 independent experiments). (**F**) ISRE reporter activity analysis of THP1-Lucia ISG cells co-cultured with CP (5 μM)-treated ENPP1-overexpressed A375 cells with or without STF-1623 (300 nM) for up to 48 hours (n=18 independent experiments). Data are presented as mean ± SD. All p values were determined by ANOVA. NS, $p > 0.05$, *$p < 0.05$, **$p < 0.01$, ***$p < 0.001$, ****$p < 0.0001$.

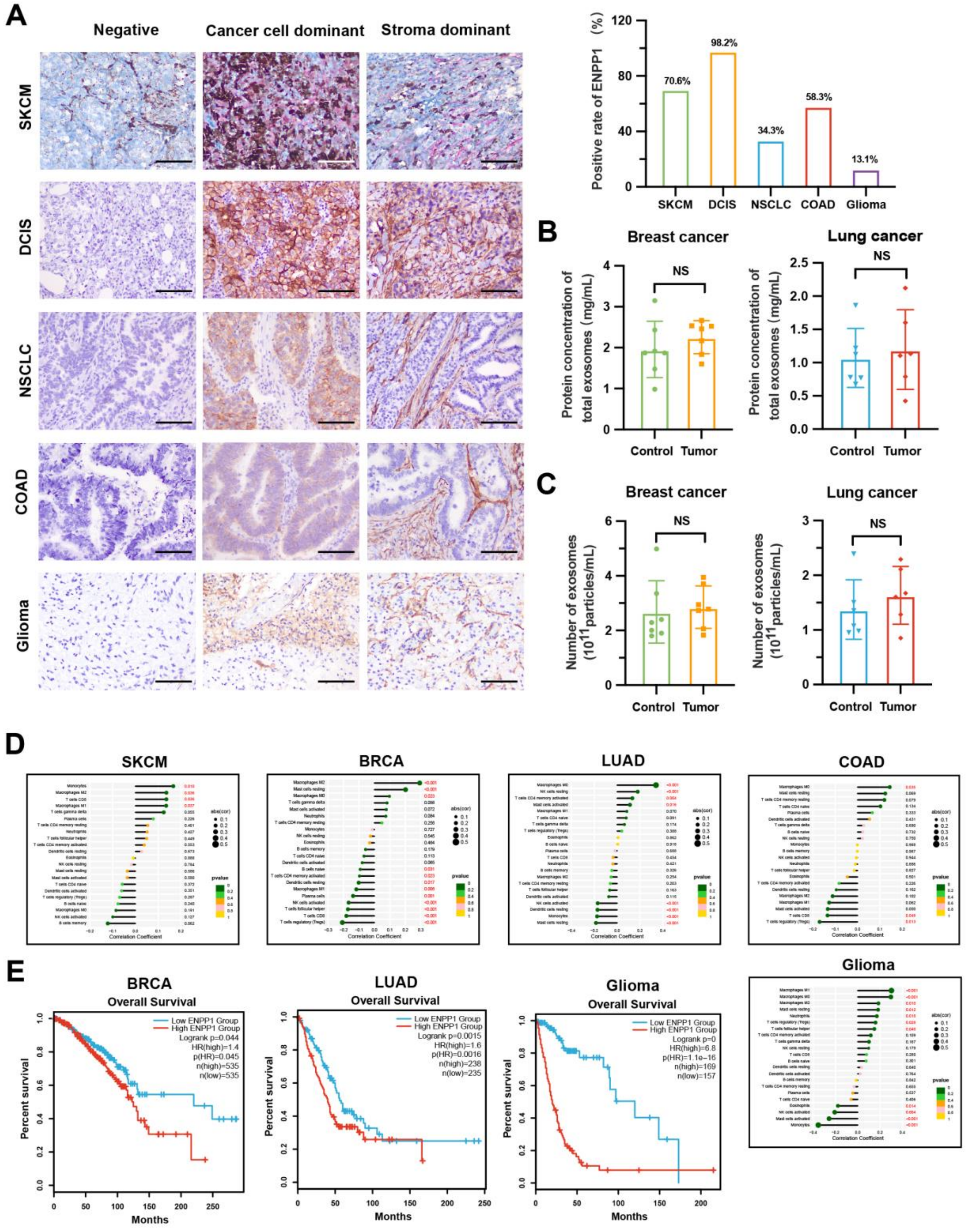


**Figure S5. Tumor tissue and tumor tissue-derived exosomes express ENPP1**. (**A**) Representative immunohistochemistry (IHC) images of human SKCM (red color means positive), DCIS (brown color means positive), NSCLC (brown color means positive), COAD (brown color means positive) and Glioma (brown color means positive) stained using anti-ENPP1 antibody (left); scale bar, 100 µm. The positive rate of ENPP1 either tumor cells or the

stroma in different tumor tissues (right). (**B**) Comparison of the total protein of exosomes between paracancer tissue and tumor tissue. (**C**) Comparison of the number of exosomes between paracancer tissue and tumor tissue. Data are presented as mean ± SD. All p values were determined by unpaired two-tailed Student's t test. NS, $p > 0.05$, *$p < 0.05$, **$p < 0.01$, ***$p < 0.001$, ****$p < 0.0001$. (**D**) Correlation between ENPP1 and immune cells in different tumors. Data from TCGA database. (**E**) Overall survival in BRCA (left), LUAD (middle), and Glioma (right) patients stratified based on their ENPP1 expression. Data from GEPIA2(cancer-pku.cn) database.

**Table S1. shRNA guide sequence**

| Gene target | shRNA | Catalog number |
|---|---|---|
| ENPP1 | shRNA1 | GCGCCTCGGCAGCGTGGCTG |
| ENPP1 | shRNA2 | CGATCGCGGCCGTTCCCCGC |
| ENPP1 | shRNA3 | CGAGAGTACTTTATAGGTGT |
